# Signal mining and analysis of adverse events associated with Isotretinoin: A 20-Year real-world pharmacovigilance study based on FAERS and EudraVigilance databases

**DOI:** 10.1371/journal.pone.0329161

**Published:** 2026-07-16

**Authors:** Qian Chen, JinYue Li, Mao Zhang, XueDong Kang, LiZhu Han, Yuan Bian, HongLi Luo

**Affiliations:** 1 Department of Pharmacy, Affiliated Hospital of Southwest Medical University, Luzhou city, Sichuan Province, China; 2 Department of Pharmacy, the First People’s Hospital of Yibin, YinBin city, Sichuan Province, China; 3 Personalized Drug Therapy Key Laboratory of Sichuan Province, Department of Pharmacy, Sichuan Academy of Medical Sciences, Sichuan Provincial People’s Hospital, Affiliated Hospital of University of Electronic Science and Technology of China, Chengdu city, Sichuan Province, China; Faculty of Medical Sciences of Minas Gerais, BRAZIL

## Abstract

**Objective:**

To systematically characterize the post-marketing safety signals of isotretinoin using real-world data from the U.S. FDA Adverse Event Reporting System (FAERS), with independent external validation in the European EudraVigilance (EV) database.

**Methods:**

Adverse event (AE) reports in FAERS from 2004Q1 to 2024Q3 were analyzed. Signal detection was conducted using four complementary disproportionality algorithms: reporting odds ratio (ROR), proportional reporting ratio (PRR), Bayesian confidence propagation neural network (BCPNN), and the multi-item gamma Poisson shrinker (MGPS). Signals concurrently detected by all four methods were defined as robust. Key findings were subsequently examined in EV as an external reference.

**Results:**

Among 50,519 patients contributing 142,160 isotretinoin-associated AE reports, 469 statistically robust signals were identified, spanning 25 system organ classes (SOCs). Signals were most concentrated in psychiatric disorders (75, 15.99%), gastrointestinal disorders (58, 12.37%), and congenital, familial, and genetic disorders (50, 10.66%). The strongest association was observed for inflammatory bowel disease (IBD; ROR = 579.14); however, temporal clustering and reporter-type profiling suggested substantial stimulated reporting, potentially driven by litigation, warranting cautious interpretation. Several high-ranking signals were not described in current product labeling, including nasal vestibulitis, hypertrophic anal papilla, and SAPHO syndrome. Pregnancy-related signals were prominent, with unintended pregnancy showing a strong signal (ROR = 91.39). External validation in EV demonstrated high concordance, supporting the robustness and reproducibility of the findings.

**Conclusions:**

Isotretinoin is associated with a broad spectrum of pharmacovigilance signals, with disproportionate representation of psychiatric, gastrointestinal, and pregnancy-related events. While multiple previously unlabelled signals emerged, their clinical relevance remains to be established. These findings underscore the need for strengthened clinical monitoring, rigorous pregnancy prevention strategies, and careful interpretation of spontaneous reporting data.

## Introduction

Isotretinoin is a first-generation, non-aromatic retinoid drug. It significantly improves acne symptoms by inhibiting sebum secretion from sebaceous glands, regulating abnormal keratinization within hair follicle ducts, exerting anti-inflammatory effects, and preventing scar formation. In 1982, isotretinoin was approved by the FDA for patients aged ≥12 years with severe refractory nodular acne. Clinically, it is also widely used for refractory or persistent moderate-to-severe acne, acne prone to scarring, or acne causing significant psychological distress [1–3].Recently, isotretinoin has frequently been utilized off-label for psoriasis, cutaneous lupus erythematosus, other skin diseases [4,5], ichthyosis, other disorders of cornification [6], and as maintenance therapy for high-risk neuroblastoma in children [7]. Currently, isotretinoin is commercially available in multiple countries worldwide, and safety concerns regarding its use remain prominent. Research indicates a high incidence of adverse reactions involving multiple organ systems, including depression, inflammatory bowel disease (IBD), liver dysfunction, and hypertriglyceridemia [8]. However, the association of isotretinoin with certain adverse reactions, such as IBD and depression, remains controversial. Therefore, a systematic analysis of large-scale, real-world data is needed to clarify potential risks. Additionally, isotretinoin is teratogenic. Prescribing information from various countries strongly advises women of childbearing age to strictly adhere to contraception while taking isotretinoin. However, the effectiveness of these recommendations requires further verification through additional real-world studies. This study also focuses on pregnancy-related events occurring during isotretinoin treatment.

The U.S. Food and Drug Administration Adverse Event Reporting System (FAERS) and the European Union’s EudraVigilance (EV) databases are based on spontaneous reporting systems. Together, they capture extensive real-world adverse event (AE) data, characterized by large sample sizes and high timeliness [9,10]. Systematic analyses of pharmacovigilance databases allow identification of potential drug-related risks and represent a cornerstone of post-marketing drug safety surveillance and regulatory pharmacovigilance.

In this study, signal detection of AEs related to isotretinoin was performed using real-world data from the FAERS database. Key findings were subsequently validated using EV as an independent external data source, enhancing the robustness of the conclusions. Reports within the EV database undergo rigorous quality control by the European Medicines Agency (EMA), including prompt removal of erroneous entries and consolidation of duplicate reports, thus ensuring high data reliability.

Through comprehensive evaluation of the safety profile of isotretinoin in real-world clinical use, this study aims to provide evidence-based insights to support clinical drug management and safe medication practices. Additionally, the results may assist regulatory authorities in identifying potential drug-related risks, issuing early warnings, and developing relevant policies.

## 1. Data and Methods

### 1.1. Data sources

This study extracted data spanning 83 quarters (from the first quarter of 2004 to the third quarter of 2024) from the FAERS database in the United States. Data are publicly accessible at https://fis.fda.gov/extensions/FPD-QDE-FAERS/FPD-QDE-FAERS.html. Definitions of FAERS database fields are available on the official FDA website. The dataset included seven subsets: demographic and administrative information (DEMO), drug/biological information (DRUG), adverse drug reaction information (REAC), patient outcome information (OUTC), report source information (RPSR), drug therapy start and end dates (THER), and indications for use or diagnosis (INDI).

EV data were obtained primarily from the publicly accessible Line Listing datasets (Stakeholder Group II, SG II) downloaded from the official EudraVigilance website. These datasets are updated weekly, and the data used in this study were retrieved on December 31, 2025. All data are accessible at https://www.adrreports.eu/en/search_subst.html.

### 1.2. Data cleaning and standardization

**Fig 1:**
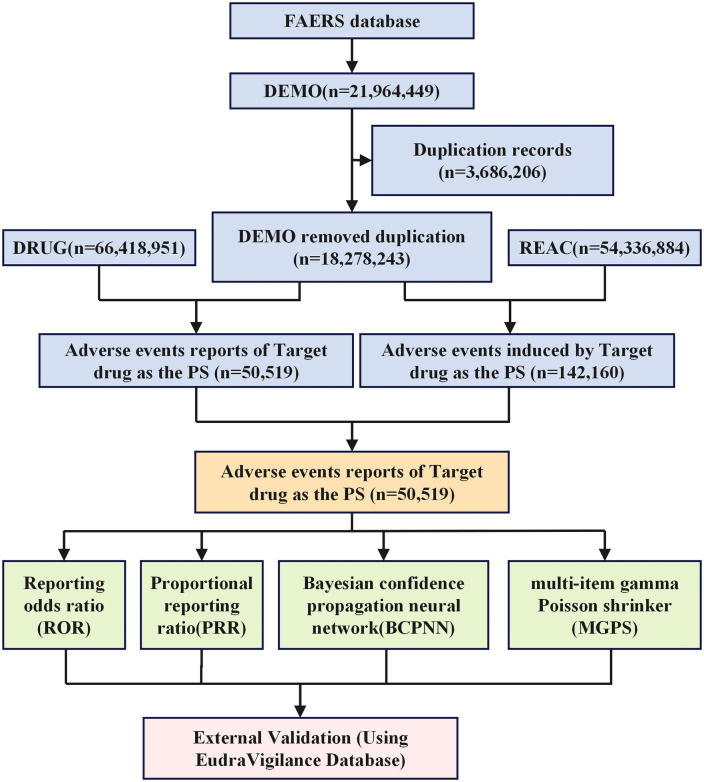
AE analysis process for Isotretinoin using the FAERS database. PS, primary suspect.

Duplicate, revoked, or deleted reports in the FAERS database required cleaning. First, duplicate reports were removed following the FDA-recommended method. The PRIMARYID, CASEID, and FDA_DT fields from the DEMO table were selected and sorted by CASEID, FDA_DT, and PRIMARYID. For reports with identical CASEID, the record with the latest FDA_DT was retained. For records with identical CASEID and FDA_DT, the report with the highest PRIMARYID was retained. Second, reports listed as deleted were removed based on CASEID. Since EV datasets from the EudraVigilance website are already deduplicated, no additional data cleaning procedures were required. Finally, AEs were classified and standardized using preferred terms (PT) and System Organ Classification (SOC) from MedDRA (version 27.1). A comprehensive flowchart of the study design is shown in Fig 1.

Data processing workflow. Data extraction and cleaning in this study were conducted primarily through automated procedures based on predefined algorithms to ensure objectivity and reproducibility. Specifically, identifying isotretinoin-related reports from FAERS subsets, deduplication procedures, and mapping of AE terms to MedDRA Preferred Terms (PTs) were all performed programmatically using SAS software (version 9.4). Upon completing the automated workflow, a manual quality control and verification step was applied. This step included logical validation of randomly selected data samples, inspection of extreme or clearly inconsistent values, and confirmation of drug–event term mappings. These manual checks aimed to detect and correct obvious anomalies, such as data entry errors, rather than to subjectively filter or reinterpret AE reports. All analyses were performed using the final dataset derived from this combined automated and manual quality verification pipeline.

### 1.3. Statistical analysis

Data processing and statistical analysis were conducted using Microsoft Excel and SAS software (version 9.4). Descriptive analysis was performed to characterize all AE reports related to isotretinoin. Currently, multiple methods are commonly used for joint mining of AE signals. This study employed four signal detection methods to enhance research credibility: ROR, PRR (based on the MHRA comprehensive criteria), BCPNN, and MGPS [11,12]. Data were classified and analyzed using a four-grid table (Table 1). Signals identified as positive by all four methods were considered risk signals associated with isotretinoin. The comprehensive criteria for signal determination were: a ≥ 3, lower limit of ROR 95% confidence interval > 1, PRR ≥ 2 and χ² ≥ 4, IC-2SD > 0, and EBGM05 > 2. Specific calculation formulas and signal criteria are provided in Table 2.

**Table 1:** Four-grid table for measures of disproportionality.

	Target AEs	Non-target AEs	Total
Target Drug	a	b	a + b
Non-target Drug	c	d	c + d
Total	a + c	b + d	N = a + b + c + d

**Table 2:** Formulas and threshold values for ROR, PRR-MHRA, BCPNN, and MGPS.

Method	Calculation Formula	Threshold
ROR	ROR = $\begin{array}{c} \frac{a/b}{c/d} \end{array}$ 95%CI for ROR = ${\text{e}}^{\text{ln(ROR)}\pm 1.96\sqrt{\left(\frac{1}{a}+\frac{1}{b}+\frac{1}{c}+\frac{1}{d}\right)}}$	a ≥ 3 and 95% CI(Lower limit) ＞ 1
PRR-MHRA	PRR = $\frac{a/(a+b)}{c/(c+d)}$ χ² = $\frac{\left(ad-bc{)}^{2}(a+b)(a+c\right)}{a+b+c+d}$	a ≥ 3, PRR Value ≥ 2 and χ² ≥ 4
BCPNN	IC = $log_{2}\frac{a(a+b+c+d)}{\left(a+b)(a+c\right)}$ IC-2SD = E(IC)-2 $\sqrt{V\left(IC\right)}$ E(IC) = $log_{2}\frac{\left(a+\gamma 11)(N+\alpha )(N+\beta \right)}{\left(N+\gamma )(a+b+\alpha_{1})(a+c+\beta_{1}\right)}$ V(IC) ≈ $\begin{array}{c} \left(\frac{1}{log2}{)}^{2}[\frac{\left(N-a+\gamma -\gamma 11\right)}{\left(a+\gamma 11\right)\left(1+N+\gamma \right)}+\frac{\left(N-a-b+\alpha -\alpha_{1}\right)}{\left(a+b+\alpha_{1})(1+N+\alpha \right)}+\frac{\left(N-a-c+\beta -\beta_{1}\right)}{\left(a+c+\beta_{1})(1+N+\beta \right)}\right] \end{array}$ γ = $\gamma_{11}\frac{\left(N+\alpha )(N+\beta \right)}{\left(a+b+\alpha_{1})(a+c+\beta_{1}\right)}$ 95%CI for IC = E(IC) ± $1.96\sqrt{V\left(IC\right)}$ Where α = α_1_ + α_2_, β = β_1_ + β_2_, N = a + b + c + d, and the value of α_1_, α_2_, β_1_, β_2_ and γ_11_ were defined as 1.	Lower limit of confidence interval（IC-2SD）＞ 0
MGPS	EBGM = $\frac{a\left(a+b+c+d\right)}{\left(a+c\right)\left(a+b\right)}$ 95%CI = $\begin{array}{c} e^{In\left(EB\text{GM}\right)\pm 1.96\sqrt{\frac{1}{a}+\frac{1}{b}+\frac{1}{c}+\frac{1}{d}}} \end{array}$	EBGM05（95% Lower limit of confidence interval）>2

#### 1.3.1. ROR method.

This method evaluates the strength of association between a specific drug and an AE by calculating the ratio of reported events. According to standardized FDA criteria, a statistically significant potential safety signal is identified when the lower bound of the 95% confidence interval (95% CI) for the ROR is greater than 1, and the number of reports exceeds three.

#### 1.3.2. PRR (based on the MHRA comprehensive criteria) method.

The PRR method assesses drug-specific risk by comparing the proportion of a specific AE reported for the target drug with that reported for all other drugs. According to UK Medicines and Healthcare products Regulatory Agency (MHRA) criteria, a safety signal is considered present when the PRR is greater than 2, the χ^2^ statistic exceeds 4, and the number of reports is at least three.

#### 1.3.3. MGPS method.

MGPS uses Bayesian statistical modeling to quantify associations between drugs and AEs. Signal strength is measured using the Empirical Bayes Geometric Mean (EBGM). A statistically significant safety signal is defined when the lower bound of the 95% CI of EBGM (EBGM05) is greater than 2.

#### 1.3.4. BCPNN method.

Based on Bayesian inference, the BCPNN method evaluates associations between drugs and AEs by calculating the Information Component (IC). According to established criteria, a potential safety signal is identified when the lower bound of the 95% CI of IC (IC–2SD) is greater than 0.

## 2. Results

### 2.1. Basic information and demographic characteristics of AE reports in the FAERS database

The total number of background patients included in this study was 18,278,243 (54,336,884 AEs), while the number of patients administered isotretinoin was 50,519 (142,160 AEs). Statistical analysis indicated a gradual annual increase in the number of AE reports associated with isotretinoin. Females accounted for 55.44%, males for 33.21%, and unknown gender for 11.35%. Patients were predominantly young, with 34.07% aged between 18 and 44 years and 19.15% younger than 18 years. The United States was the primary reporting country, contributing 80.14% of reports. Physicians (33.47%) and consumers (29.52%) were the main reporters. Specific details are shown in Table 3.

**Table 3:** Basic information of AE reports related to isotretinoin in the FAERS database.

Indicators	No. of AEs (%)
**Gender**	
Female	28006 (55.44)
Male	16779 (33.21)
Unknown	5734 (11.35)
**Age**	
<18 years old	9676 (19.15)
18-44 years old	17210 (34.07)
45-64 years old	1226 (2.43)
≥65 years old	239 (0.47)
Unknown	22168 (43.88)
**Reporting year**	
2004	1153 (2.28)
2005	1292 (2.56)
2006	1508 (2.99)
2007	1383 (2.74)
2008	1795 (3.55)
2009	1290 (2.55)
2010	1565 (3.10)
2011	2650 (5.25)
2012	2686 (5.32)
2013	3098 (6.13)
2014	2897 (5.73)
2015	2658 (5.26)
2016	2123 (4.20)
2017	3242 (6.42)
2018	2260 (4.47)
2019	2457 (4.86)
2020	3320 (6.57)
2021	3131 (6.20)
2022	3729 (7.38)
2023	3292 (6.52)
2024（1–3Quarterlydata）	2990 (5.92)
**Reporters**	
Doctor	16911 (33.47)
Consumer	14912 (29.52)
Lawyer	5809 (11.50)
Pharmacist	4263 (8.44)
Other health professionals	6141 (12.16)
Unknown	2483 (4.91)
**Reporting country**	
America	40486 (80.14)
Britain	1047 (2.07)
Brazil	951 (1.88)
Canada	517 (1.02)
France	315 (0.62)
Other country	7203 (14.26)

### 2.2. Signal intensity of AEs related to isotretinoin in the FAERS database

This study identified 469 positive AE signals related to isotretinoin through the joint application of four methods. The AE signals detected by each method were largely consistent (Table 4). These results generally aligned with AE occurrences documented in isotretinoin prescribing information, confirming the credibility of the analytical method. Ranked in descending order by ROR, the top ten AEs were IBD, gastrointestinal injury, acne fulminans, ulcerative proctitis, premature epiphyseal closure, nasal vestibulitis, induced complete abortion, selective abortion, xerosis, and epiphyseal injury. Several AEs not documented in the prescribing information were identified, including nasal vestibulitis, hypertrophic anal papilla, neonatal neuroblastoma, diverticular hernia, SAPHO syndrome, somatic delusional disorder, hypersomnia-bulimia syndrome, and hemihypertrophy. Specific details are shown in S1 and S5 Tables.

**Table 4:** The top 30 AE signals related to isotretinoin in the FAERS database.

PTs	No. of AEs	ROR (95% CI)	PRR (Chi-Square)	IC (IC-2SD)	EBGM (EBGM05)
Inflammatory bowel disease	5278	579.14(554.95-604.39)	557.68(1191034)	7.83(7.72)	227.03(217.54)
Gastrointestinal injury	1260	412.80(381.18-447.04)	409.15(247454)	7.63(7.32)	197.86(182.71)
Acne fulminans	75	321.42(236.39-437.04)	321.26(12994.3)	7.45(5.33)	174.80(128.56)
Proctitis ulcerative	193	241.56(201.70-289.30)	241.23(28278.8)	7.21(6.15)	148.13(123.69)
Epiphyses premature fusion	98	221.22(172.47-283.74)	221.06(13588.9)	7.13(5.52)	140.29(109.38)
Nasal vestibulitis*	30	219.98(140.35-344.79)	219.94(4146.20)	7.13(4.06)	139.84(89.22)
Abortion induced complete	11	209.69(100.47-437.64)	209.67(1473.84)	7.08(2.49)	135.63(64.98)
Selective abortion	59	195.66(142.95-267.82)	195.58(7548.89)	7.02(4.93)	129.60(94.69)
Xerosis	339	183.75(161.40-209.18)	183.31(41508.4)	6.96(6.33)	124.11(109.02)
Epiphyseal injury	9	163.39(74.83-356.76)	163.38(1016.74)	6.84(2.16)	114.67(52.52)
Lip dry	1806	143.68(136.09-151.70)	141.87(184126)	6.70(6.54)	103.66(98.19)
Pityriasis alba	3	127.08(34.40-469.41)	127.07(281.44)	6.58(0.30)	95.56(25.87)
Hypertrophic anal papilla*	5	119.14(43.64-325.21)	119.13(446.25)	6.51(1.18)	91.01(33.34)
Neonatal neuroblastoma*	3	114.37(31.47-415.58)	114.37(259.33)	6.46(0.31)	88.21(24.27)
Anomaly of middle ear congenital	4	108.92(35.85-330.92)	108.92(332.67)	6.41(0.80)	84.94(27.96)
Diverticular hernia*	3	103.97(29.01-372.69)	103.97(240.38)	6.36(0.32)	81.91(22.85)
Childhood depression	3	103.97(29.01-372.69)	103.97(240.38)	6.36(0.32)	81.91(22.85)
Anotia	18	100.92(60.03-169.68)	100.91(1407.92)	6.32(3.22)	80.00(47.58)
Cheilosis	9	98.04(47.12-203.95)	98.03(687.55)	6.29(2.15)	78.18(37.58)
Acne conglobata	13	95.31(51.90-175.03)	95.31(970.49)	6.26(2.73)	76.44(41.63)
Congenital cerebellar agenesis	8	95.31(43.92-206.84)	95.31(597.23)	6.26(1.96)	76.44(35.23)
Unintended pregnancy	1788	91.39(86.78-96.26)	90.26(127634)	6.19(6.06)	73.17(69.48)
Dermatitis papillaris capillitii	3	87.98(25.07-308.74)	87.97(209.59)	6.16(0.33)	71.67(20.42)
Abortion induced	1600	87.93(83.26-92.86)	86.95(110710)	6.15(6.01)	70.99(67.22)
Drug exposure before pregnancy	168	81.58(69.05-96.37)	81.48(11003.5)	6.07(5.35)	67.31(56.98)
SAPHO syndrome*	36	81.23(56.68-116.40)	81.21(2351.09)	6.07(4.07)	67.12(46.84)
Delusional disorder, somatic type *	3	76.25(22.07-263.38)	76.24(185.64)	5.99(0.34)	63.70(18.44)
Hypersomnia-bulimia syndrome*	4	72.62(24.93-211.55)	72.61(237.31)	5.93(0.82)	61.16(20.99)
Chapped lips	580	67.64(61.92,73.90)	67.37(32230.3)	5.84(5.58)	57.40(52.54)
Hemihypertrophy*	3	63.54(18.72,215.71)	63.54(158.28)	5.77(0.35)	54.60(16.08)

### 2.3 Classification of system organs involved in AE signals related to isotretinoin in the FAERS database

AE reports involved 27 organ classes according to the SOC classification. Specific details are shown in S2 Table. The highest number of reports occurred in Gastrointestinal disorders (31,138 cases, 21.90%), followed by Psychiatric disorders (23,094 cases, 16.25%) and Injury, poisoning, and procedural complications (13,973 cases, 9.83%). Signal intensities for SOC-related AEs associated with isotretinoin are presented in Table 5.

**Table 5:** Distribution of SOC involved in isotretinoin-related AEs and cumulative signal intensity for each SOC in the FAERS database.

SOC	AE signals		All AEs					
	No. of AEs	Proportion (%)	Reporting No. of AEs	Proportion (%)	ROR (95% CI)	PRR (Chi-Square)	IC (IC-2SD)	EBGM (EBGM05)
Psychiatric disorders	75	15.99	23094	16.25	3.25(3.21,3.30)	2.89(29982.1)	1.52(1.50)	2.87(2.83)
Gastrointestinal disorders	58	12.37	31138	21.9	3.03(2.99,3.07)	2.59(32867.4)	1.36(1.35)	2.57(2.54)
Congenital, familial and genetic disorders	50	10.66	914	0.64	2.14(2.01,2.29)	2.13(549.14)	1.09(0.99)	2.13(1.99)
Investigations	43	9.17	8059	5.67	0.92(0.90,0.94)	0.92(56.93)	−0.12(−0.15)	0.92(0.90)
Skin and subcutaneous tissue disorders	37	7.89	8911	6.27	1.18(1.15,1.20)	1.17(221.79)	0.22(0.19)	1.17(1.14)
Infections and infestations	30	6.4	4257	2.99	0.56(0.54,0.58)	0.57(1441.17)	−0.81(−0.85)	0.57(0.55)
Injury, poisoning and procedural complications	29	6.18	13973	9.83	0.95(0.93,0.96)	0.95(38.85)	−0.07(−0.10)	0.95(0.93)
Pregnancy, puerperium and perinatal conditions	25	5.33	6453	4.54	11.27(10.99,11.56)	10.81(56092.8)	3.40(3.36)	10.54(10.27)
Musculoskeletal and connective tissue disorders	20	4.26	7576	5.33	1.03(1.01,1.05)	1.03(6.56)	0.04(0.01)	1.03(1.01)
Reproductive system and breast disorders	16	3.41	1420	1	1.12(1.06,1.18)	1.12(18.21)	0.16(0.09)	1.12(1.06)
Neoplasms benign, malignant and unspecified (incl cysts and polyps)	14	2.99	802	0.56	0.21(0.20,0.22)	0.21(2384.32)	−2.22(−2.33)	0.21(0.20)
Eye disorders	13	2.77	3345	2.35	1.19(1.15,1.23)	1.18(96.09)	0.24(0.19)	1.18(1.14)
Surgical and medical procedures	13	2.77	3039	2.14	1.61(1.55,1.67)	1.59(678.91)	0.67(0.62)	1.59(1.54)
General disorders and administration site conditions	11	2.35	12845	9.04	0.47(0.46,0.48)	0.52(6989.88)	−0.95(−0.98)	0.52(0.51)
Social circumstances	7	1.49	514	0.36	0.78(0.71,0.85)	0.78(32.71)	−0.36(−0.49)	0.78(0.71)
Nervous system disorders	6	1.28	6106	4.3	0.48(0.47,0.49)	0.50(3247.97)	−0.99(−1.02)	0.50(0.49)
Renal and urinary disorders	5	1.07	802	0.56	0.29(0.27,0.31)	0.30(1375.87)	−1.76(−1.86)	0.30(0.28)
Blood and lymphatic system disorders	4	0.85	1105	0.78	0.46(0.43,0.48)	0.46(709.63)	−1.12(−1.20)	0.46(0.43)
Metabolism and nutrition disorders	4	0.85	1129	0.79	0.36(0.34,0.38)	0.37(1271.72)	−1.45(−1.54)	0.37(0.35)
Respiratory, thoracic and mediastinal disorders	3	0.64	2932	2.06	0.43(0.41,0.44)	0.44(2219.46)	−1.19(−1.24)	0.44(0.42)
Ear and labyrinth disorders	2	0.43	543	0.38	0.88(0.81,0.96)	0.88(9.12)	−0.19(−0.31)	0.88(0.81)
Vascular disorders	1	0.21	749	0.53	0.24(0.23,0.26)	0.25(1766.02)	−2.02(−2.12)	0.25(0.23)
Endocrine disorders	1	0.21	162	0.11	0.45(0.38,0.52)	0.45(109.99)	−1.15(−1.38)	0.45(0.39)
Immune system disorders	1	0.21	492	0.35	0.31(0.29,0.34)	0.31(743.46)	−1.67(−1.80)	0.31(0.29)
Hepatobiliary disorders	1	0.21	612	0.43	0.47(0.43,0.50)	0.47(371.56)	−1.09(−1.21)	0.47(0.43)
Cardiac disorders	0	0	662	0.47	0.17(0.16,0.19)	0.18(2622.88)	−2.50(−2.61)	0.18(0.16)
Product issues	0	0	526	0.37	0.23(0.21,0.25)	0.23(1386.85)	−2.12(−2.25)	0.23(0.21)
Total	469	100	142160	100.00				

System organs are sorted in descending order by the number of AE signals.

Positive AE signals involved 25 SOC classes. Ranked by the number of signals, the top three were Psychiatric disorders (75, 15.99%), Gastrointestinal disorders (58, 12.37%), and Congenital, familial, and genetic disorders (50, 10.66%).

Four methods were applied to detect cumulative signals across SOC classes. Positive cumulative signals were identified in three SOC classes. Ranked by descending ROR values, these were Pregnancy, puerperium, and perinatal conditions (ROR = 11.27, 95% CI lower limit = 10.99), Psychiatric disorders (ROR = 3.25, 95% CI lower limit = 3.21), and Gastrointestinal disorders (ROR = 3.03, 95% CI lower limit = 2.99).

### 2.4. Onset Time of AEs in the FAERS database

**Fig 2:**
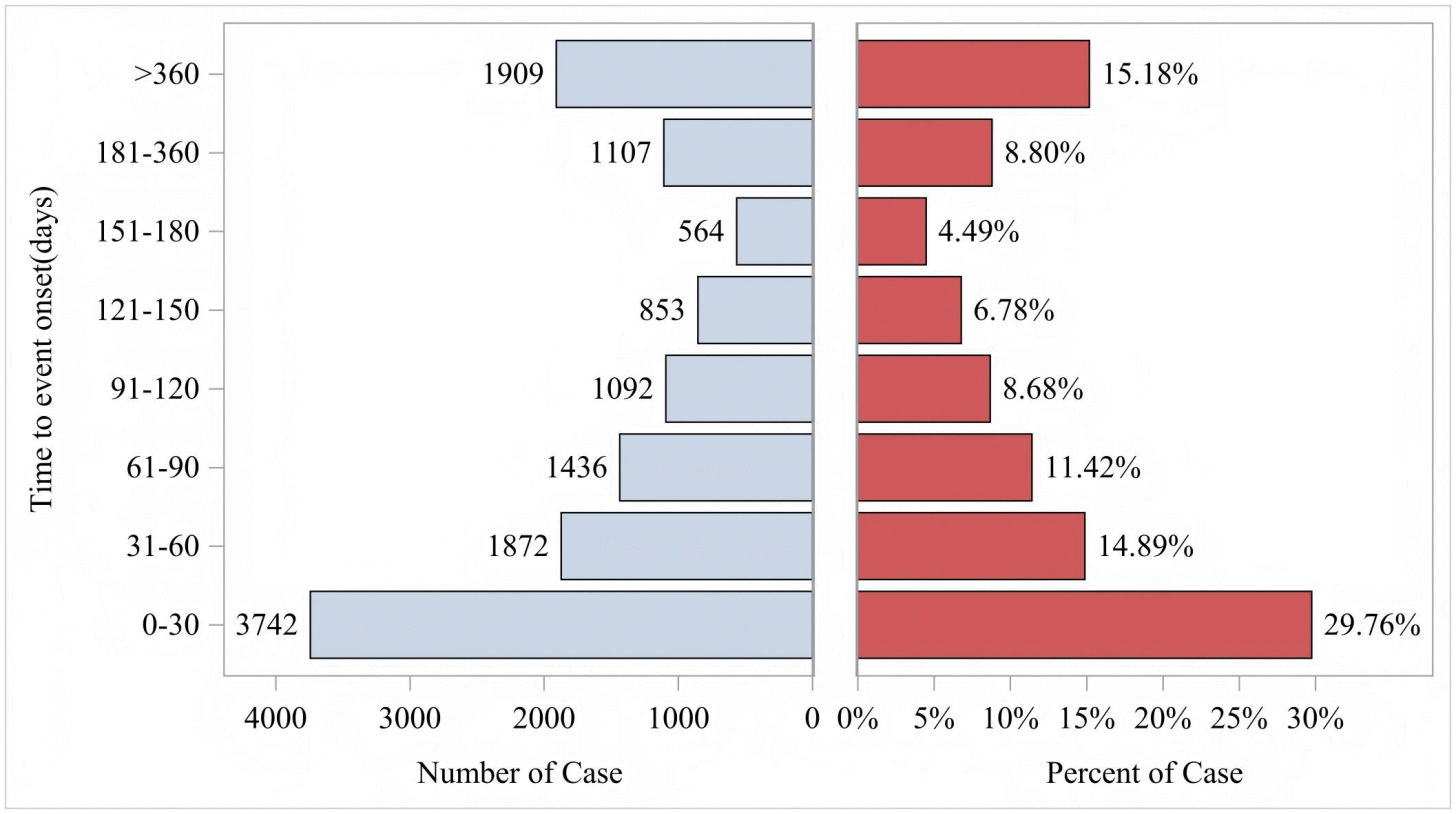
Time-to-event report distribution of AE reports in the FAERS database.

**Fig 3:**
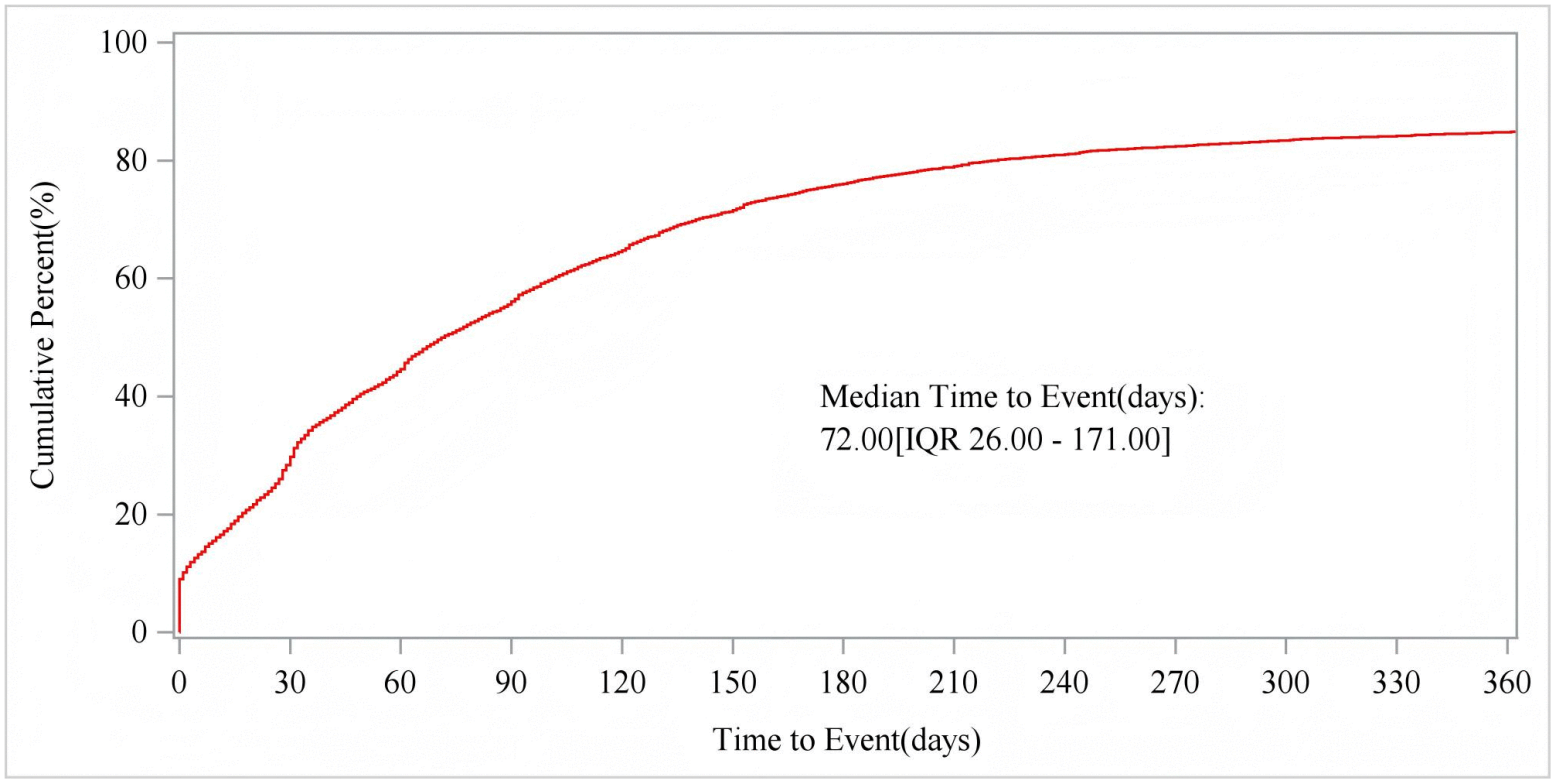
Cumulative incidence of AEs in the FAERS database.

After excluding reports with inaccurate, missing, or unknown onset times, 12,575 AEs associated with isotretinoin provided valid onset-time data. The median AE onset time was 72 days (interquartile range [IQR]: 26–171 days), with more than half occurring within the first three months of treatment (0–30 days: n=3,742, 29.76%; 31–60 days: n=1,872, 14.89%; 61–90 days: n=1,436, 11.42%). The incidence of AEs after six months was considerable, showing an increasing trend, and accounting for 23.98% of total cases (181–360 days: n=1,107, 8.80%; >360 days: n=1,909, 15.18%). The timeline of these events is illustrated in Fig 2, and the cumulative incidence curve of AEs is presented in Fig 3.

### 2.5. Result of external validation

#### 2.5.1. Basic information and demographic characteristics of AE reports in the EV database.

The EV database included 11,906,424 background patients, accounting for 50,987,250 reported AEs. Among these, 34,920 patients had reports involving isotretinoin, corresponding to 124,146 AE occurrences. The overall data volume was comparable to that of the FAERS database.

Regarding sex distribution, females accounted for 50.87% of cases, males for 43.33%, and unspecified gender for 5.80%, closely mirroring FAERS data. Most reports involved individuals aged 18–64 years (49.45%), followed by those younger than 18 years (23.62%). This age distribution aligned with observations in the FAERS database, indicating that reported AEs were predominantly concentrated among adolescents and young adults. Detailed data are presented in Table 6.

**Table 6:** Basic information of AE reports related to isotretinoin in the EV database.

Indicators	No. of AEs (%)
**Gender**	
Female	17764(50.87)
Male	15130(43.33)
Not Specified	2026(5.80)
**Age**	
<18	8249(23.62)
18-64	17269(49.45)
≥65	137(0.39)
Not Specified	9265(26.53)
**Reportingyear**	
2004	1004(2.88)
2005	738(2.11)
2006	943(2.70)
2007	979(2.80)
2008	8178(23.42)
2009	879(2.52)
2010	1004(2.88)
2011	1835(5.25)
2012	2472(7.08)
2013	2777(7.95)
2014	2095(6.00)
2015	1302(3.73)
2016	841(2.41)
2017	748(2.14)
2018	1004(2.88)
2019	1270(3.64)
2020	1035(2.96)
2021	965(2.76)
2022	1730(4.95)
2023	923(2.64)
2024	1349(3.86)
2025	849(2.43)
**Reporters**	
Healthcare Professional	20465(58.61)
Non Healthcare Professional	14360(41.12)
Not Specified	95(0.27)
**Reporting country**	
European Economic Area	12938(37.05)
Non European Economic Area	21981(62.95)
Not Specified	1(0.00)

### 2.5.2. Signal intensity of AEs related to isotretinoin in the EV database

In the EV database, 550 AE signals related to isotretinoin were jointly identified as positive by all four signal detection methods. The AE signals detected by individual methods were largely consistent (Table 7) and generally matched AEs documented in the product label. Specific details are shown in S3 Table. When ranked in descending order by ROR values, the top ten signals were urinary meatitis, IBD, acne fulminans, congenital thymus absence, gastrointestinal injury, epiphyseal premature fusion, hypertrophic anal papilla, ulcerative proctitis, anotia, and lupus miliaris disseminatus faciei. Most of these signals were consistent with those identified in the FAERS database.

**Table 7:** The top 30 AE signals related to isotretinoin in the EV database.

PTs	No. of AEs	ROR (95% CI)	PRR (Chi-Square)	IC (IC-2SD)	EBGM (EBGM05)
Urinary meatitis	4	1638.87(183.17-14663.5)	1638.82(1309.45)	8.36(0.55)	328.56(36.72)
IBD	6300	830.72(796.06-866.89)	788.61(1694660)	8.08(7.97)	270.28(259.01)
Acne fulminans	207	606.79(489.62-751.99)	605.78(50425.4)	7.94(6.56)	245.00(197.69)
Congenital thymus absence	4	546.29(122.26-2440.93)	546.27(933.04)	7.87(0.65)	234.69(52.52)
Gastrointestinal injury	1157	530.46(486.15-578.82)	525.53(265365)	7.85(7.48)	230.78(211.50)
Epiphyses premature fusion	98	478.36(357.41-640.25)	477.99(21529.5)	7.79(5.74)	221.15(165.23)
Hypertrophic anal papilla	8	273.15(111.65-668.25)	273.14(1301.47)	7.36(1.95)	164.28(67.15)
Proctitis ulcerative	213	264.90(222.96-314.73)	264.45(33973.7)	7.33(6.29)	161.10(135.60)
Anotia	24	213.80(130.51-350.24)	213.76(3339.81)	7.14(3.74)	140.81(85.96)
Lupus miliaris disseminatus faciei	3	204.86(51.23-819.14)	204.85(405.71)	7.10(0.26)	136.90(34.24)
Perirectal abscess	157	126.53(105.79-151.34)	126.37(14924.5)	6.60(5.66)	96.82(80.95)
Pouchitis	79	119.07(92.68-152.98)	118.99(7162.92)	6.53(5.07)	92.44(71.95)
Congenital cerebellar agenesis	9	111.75(53.47-233.53)	111.74(776.06)	6.46(2.17)	88.01(42.11)
Nasal vestibulitis	30	109.77(73.36-164.24)	109.74(2549.61)	6.44(3.95)	86.77(57.99)
Xerosis	224	98.33(84.98-113.78)	98.15(17377.9)	6.31(5.67)	79.38(68.60)
Anomaly of middle ear congenital	4	96.40(32.44-286.51)	96.40(305.71)	6.29(0.82)	78.23(26.32)
Night blindness	148	94.86(79.32-113.43)	94.74(11149.6)	6.27(5.41)	77.14(64.50)
Chapped lips	432	93.72(84.41-104.06)	93.40(32159.6)	6.25(5.87)	76.25(68.67)
Pregnancy	1663	91.02(86.29-96.01)	89.82(119816)	6.21(6.07)	73.85(70.01)
Rectal fissure	62	83.05(63.21-109.13)	83.01(4177.17)	6.11(4.66)	69.19(52.66)
Congenital optic nerve anomaly	4	81.94(28.01-239.75)	81.94(266.51)	6.10(0.82)	68.45(23.40)
Pityriasis alba	3	81.94(23.72-283.06)	81.94(199.88)	6.10(0.35)	68.45(19.82)
Pregnancy of partner	35	80.13(55.78-115.12)	80.11(2287.09)	6.07(4.04)	67.17(46.76)
Lip dry	1919	77.51(73.80-81.40)	76.32(120281)	6.01(5.89)	64.50(61.41)
Meibomian gland dysfunction	44	77.40(56.08-106.82)	77.37(2789.96)	6.03(4.28)	65.24(47.27)
Cheilosis	9	76.82(37.70-156.57)	76.82(567.15)	6.02(2.14)	64.85(31.82)
Depression	3	76.82(22.38-263.65)	76.82(189.05)	6.02(0.35)	64.85(18.89)
Anorectal stenosis	4	74.49(25.67-216.18)	74.49(245.40)	5.98(0.83)	63.19(21.77)
Enterocolonic fistula	11	73.89(38.88-140.42)	73.88(670.02)	5.97(2.45)	62.75(33.02)
Colitis ulcerative	4901	73.19(70.97-75.48)	70.34(286099)	5.91(5.85)	60.18(58.35)

AE signals are sorted by descending ROR values.

### 2.5.3. Classification of system organs involved in AE signals related to isotretinoin in the EV database

In the EV database, reported AEs spanned 27 System Organ Classes (SOCs). Specific details are shown in S4 Table. The majority of AE reports occurred in gastrointestinal disorders (34,012 cases, 27.40%) and psychiatric disorders (19,859 cases, 16.00%), a distribution broadly consistent with FAERS data. In contrast, the category of injury, poisoning, and procedural complications, ranked third in FAERS, ranked seventh in the EV database (6,413 cases, 5.17%).

Positive AE signals in the EV database involved 25 SOCs. Ranked by the number of signals, the top three SOCs were psychiatric disorders (73 signals, 13.27%), congenital, familial, and genetic disorders (71 signals, 12.91%), and gastrointestinal disorders (66 signals, 12.00%). These findings were again broadly consistent with those from FAERS.

Based on cumulative SOC-level signals jointly detected by all four methods, four SOCs demonstrated statistically significant positive signals. Ranked by descending ROR values, these were pregnancy, puerperium, and perinatal conditions (ROR = 5.70, 95% CI lower limit = 5.50), gastrointestinal disorders (ROR = 3.74, 95% CI lower limit = 3.69), psychiatric disorders (ROR = 3.57, 95% CI lower limit = 3.51), and congenital, familial, and genetic disorders (ROR = 3.45, 95% CI lower limit = 3.26). In contrast, cumulative signals for congenital, familial, and genetic disorders were not positive in the FAERS database. Findings for the other SOCs were largely concordant between the two databases. Detailed data are presented in Table 8.

**Table 8:** Distribution of SOC involved in isotretinoin-related AEs and cumulative signal intensity for each SOC in the EV database.

SOC	AE signals		All AEs					
	No. of AEs	Proportion (%)	Reporting No. of AEs	Proportion (%)	ROR (95% CI)	PRR (Chi-Square)	IC (IC-2SD)	EBGM (EBGM05)
Psychiatric disorders	73	13.27	19859	16.00	3.57(3.51,3.62)	3.16(30592.3)	1.65(1.63)	3.14(3.09)
Congenital, familial and genetic disorders	71	12.91	1234	0.99	3.45(3.26,3.65)	3.43(2107.46)	1.77(1.68)	3.41(3.22)
Gastrointestinal disorders	66	12.00	34012	27.40	3.74(3.69,3.79)	2.99(49233.9)	1.57(1.56)	2.97(2.94)
Skin and subcutaneous tissue disorders	58	10.55	9190	7.40	1.21(1.19,1.24)	1.20(321.98)	0.26(0.23)	1.20(1.17)
Infections and infestations	42	7.64	3852	3.10	0.60(0.58,0.62)	0.61(978.11)	−0.70(−0.75)	0.62(0.60)
Investigations	30	5.45	7268	5.85	0.88(0.86,0.90)	0.89(110.02)	−0.17(−0.21)	0.89(0.87)
Musculoskeletal and connective tissue disorders	30	5.45	7137	5.75	0.97(0.95,0.99)	0.97(5.92)	−0.04(−0.08)	0.97(0.95)
Pregnancy, puerperium and perinatal conditions	30	5.45	3384	2.73	5.70(5.50,5.90)	5.57(12576.6)	2.46(2.41)	5.51(5.32)
Eye disorders	25	4.55	3834	3.09	1.75(1.69,1.80)	1.72(1182.60)	0.78(0.74)	1.72(1.67)
Injury, poisoning and procedural complications	24	4.36	6413	5.17	0.73(0.71,0.75)	0.74(603.00)	−0.42(−0.46)	0.74(0.73)
Reproductive system and breast disorders	23	4.18	1589	1.28	1.10(1.04,1.15)	1.10(13.41)	0.13(0.06)	1.10(1.04)
Neoplasms benign, malignant and unspecified (incl cysts and polyps)	17	3.09	868	0.70	0.25(0.23,0.27)	0.26(1936.25)	−1.97(−2.06)	0.26(0.24)
General disorders and administration site conditions	11	2.00	7415	5.97	0.34(0.33,0.35)	0.38(9027.93)	−1.40(−1.44)	0.38(0.37)
Blood and lymphatic system disorders	8	1.45	1630	1.31	0.42(0.40,0.44)	0.43(1303.67)	−1.23(−1.30)	0.43(0.41)
Surgical and medical procedures	6	1.09	1105	0.89	1.08(1.02,1.15)	1.08(6.40)	0.11(0.02)	1.08(1.02)
Nervous system disorders	5	0.91	5762	4.64	0.47(0.46,0.49)	0.50(3220.59)	−1.01(−1.04)	0.50(0.49)
Respiratory, thoracic and mediastinal disorders	5	0.91	2940	2.37	0.48(0.46,0.50)	0.49(1629.91)	−1.03(−1.08)	0.49(0.47)
Metabolism and nutrition disorders	5	0.91	1413	1.14	0.49(0.46,0.52)	0.50(743.10)	−1.01(−1.09)	0.50(0.47)
Renal and urinary disorders	5	0.91	978	0.79	0.31(0.29,0.33)	0.31(1517.08)	−1.68(−1.77)	0.31(0.29)
Ear and labyrinth disorders	5	0.91	680	0.55	1.06(0.99,1.15)	1.06(2.54)	0.09(−0.02)	1.06(0.99)
Social circumstances	4	0.73	241	0.19	0.55(0.48,0.62)	0.55(89.97)	−0.87(−1.05)	0.55(0.48)
Hepatobiliary disorders	3	0.55	983	0.79	0.58(0.54,0.62)	0.58(300.48)	−0.78(−0.87)	0.58(0.55)
Vascular disorders	2	0.36	976	0.79	0.32(0.30,0.34)	0.33(1376.55)	−1.61(−1.70)	0.33(0.31)
Immune system disorders	1	0.18	337	0.27	0.18(0.16,0.20)	0.19(1221.53)	−2.43(−2.58)	0.19(0.17)
Endocrine disorders	1	0.18	266	0.21	0.57(0.51,0.64)	0.57(85.97)	−0.81(−0.98)	0.57(0.51)
Cardiac disorders	0	0	734	0.59	0.20(0.19,0.22)	0.21(2313.09)	−2.28(−2.38)	0.21(0.19)
Product issues	0	0	46	0.04	0.06(0.05,0.08)	0.06(634.85)	−3.97(−4.36)	0.06(0.05)
Total	550	100.00	124146	100.00				

System organs are sorted in descending order by the number of AE signals.

## 3. Discussion

This study systematically analyzed AE signals related to isotretinoin using the FAERS spontaneous reporting system and four statistical methods. It is important to note that spontaneous reporting data only indicate statistical associations between drugs and AEs, not causality. Signal detection aims to identify potential risk signals to guide future clinical research, regulatory decisions, and clinical monitoring, rather than confirming direct causation. Therefore, the following discussion should be framed within the context of “association,” avoiding overinterpretation.

### 3.1. Basic characteristics of AE reports related to isotretinoin

Patients included in isotretinoin-related AE reports were predominantly young adults and minors, with females constituting the majority. This distribution may be related to the higher incidence of acne in post-adolescent females compared to males, and the significantly greater proportion of females seeking medical treatment. Previous studies indicate that acne incidence is roughly equal between genders during adolescence, but prevalence becomes higher among females after adolescence [13,14]. Due to factors including appearance-related anxiety, sociocultural pressures, and physiological characteristics [15], females are more inclined to seek professional treatment [16], such as prescription medications or long-term therapy. In contrast, males typically seek medical attention only in severe cases.

### 3.2. Characteristics of isotretinoin-related AEs involving SOC

This study identified AE signals involving 25 SOC categories, suggesting isotretinoin-related AEs potentially affect multiple organ systems. Prior research demonstrated that isotretinoin treatment affects nearly all human body systems, resulting in diverse AEs [17]. This conclusion aligns closely with findings from our study. Therefore, comprehensive monitoring and timely interventions are essential in clinical practice. SOC categories with high signal counts and AE reports included primarily psychiatric disorders, gastrointestinal disorders, congenital, familial, and genetic disorders, investigations, and skin and subcutaneous tissue disorders. These findings are largely consistent with previous studies [18] and with isotretinoin prescribing information, confirming the feasibility and reliability of the methods used.

AE signals and reports for psychiatric and gastrointestinal disorders ranked highest, significantly exceeding other SOC categories. These findings indicate that AEs in these categories occur most frequently clinically. Prominent PT signals within psychiatric disorders (ranked by descending ROR) included childhood depression, somatic delusional disorder, hypersomnia-bulimia syndrome, purging behaviors, and emotional changes associated with eating disorders. Prominent PT signals within gastrointestinal disorders (ranked by descending ROR) included IBD, ulcerative proctitis, cheilosis, hypertrophic anal papilla, and diverticular hernia.

Analysis of cumulative SOC signals revealed that pregnancy, puerperium, and perinatal conditions exhibited the strongest signals across all four detection methods. This SOC category accounted for 6,453 reported events, of which 4,641 (71.92%) were pregnancy-related, presenting robust positive signals. These results highlight the necessity for careful monitoring of pregnancy-related events during isotretinoin therapy.

### 3.3. Onset time of isotretinoin-associated AEs

The study indicated a median onset time of AEs of 72 days after initiating isotretinoin treatment. The majority of events occurred within the first three months of therapy. Furthermore, the incidence of AEs showed an upward trend after six months of treatment. Thus, clinical monitoring for AEs is particularly important during the initial three months and after six months of continuous isotretinoin therapy to ensure patient safety.

### 3.4. AE signals of key concern

#### 3.4.1. Isotretinoin and AE signals related to psychiatric disorders.

Currently, the association between isotretinoin and psychiatric disorders remains controversial. Studies suggest that isotretinoin may elevate risks of anxiety and depression by reducing serotonin levels, increasing neuronal apoptosis, regulating gene expression, affecting neural plasticity, or activating the HPA axis [19]. However, other research indicates that isotretinoin is primarily prescribed for moderate-to-severe acne, a condition inherently associated with a higher prevalence of psychiatric disorders [20], particularly among adolescents and young adults. Patients often experience depression, anxiety, and suicidal tendencies due to changes in physical appearance and social anxiety [21]. Thus, patients prescribed isotretinoin inherently face a higher risk of psychiatric disorders. The influence of acne itself must be comprehensively considered when investigating psychiatric side effects related to isotretinoin [22]. Recent large-scale cohort studies and meta-analyses have not definitively confirmed a causal relationship [23].

It should be noted that psychiatric signals detected in this study represent statistical associations only and do not imply direct causation by isotretinoin. Acne itself and its associated psychosocial stress may serve as confounding factors for psychiatric symptoms. Future prospective cohort or case-control studies are required to further investigate causality.

Although existing evidence does not sufficiently establish causality between isotretinoin and psychiatric disorders, the association cannot be entirely ruled out. Monitoring of psychiatric AEs is recommended in both domestic and international prescribing information for isotretinoin. This study identified strong psychiatric AE signals based on extensive real-world data from isotretinoin users, warranting serious attention. Clinically, mental health screening prior to isotretinoin therapy is advised. Psychological status should be routinely monitored throughout treatment. Personalized management plans should be implemented for high-risk patients. Psychological support and regular follow-up are necessary during therapy to mitigate psychiatric risks. Education on medication for both patients and families must be strengthened. Patients displaying psychiatric symptoms should promptly seek medical care. Multidisciplinary management approaches should be improved, including collaboration with psychiatric or psychological specialists as needed.

#### 3.4.2. Isotretinoin and AE signals related to IBD.

This study identified an exceptionally strong signal for inflammatory bowel disease (IBD), with a reporting odds ratio (ROR) of 579.14, ranking first among all detected signals and second in the number of reported cases, indicating an unusually high statistical strength. Given the extraordinary magnitude of the IBD signal, a detailed analysis of reporting patterns was conducted to better contextualize this finding.

In total, 5,278 IBD reports were identified, of which 4,483 (84.94%) were submitted by legal professionals. Temporal analysis further revealed a significant clustering of reports between 2009 and 2014, during which 4,526 cases (85.75%) occurred. In contrast, since 2018, fewer than 10 IBD reports have been submitted annually. This markedly skewed distribution in reporter type and reporting period strongly suggests non-random reporting dynamics.

To further elucidate the potential reasons for the strong isotretinoin-IBD association observed, the broader regulatory and legal context was examined. Following 2009, multiple high-profile lawsuits were filed in the United States against isotretinoin manufacturers, coinciding with a marked surge in IBD reports from 2009–2012. Previous investigations indicated that a substantial proportion of FAERS reports linking isotretinoin to IBD originated from law firms, highlighting potential litigation-driven stimulated reporting [24, 25]. The reporting patterns observed in the present study closely align with known timelines of litigation activity in the United States. These results are consistent with previous studies documenting attorney-driven waves of isotretinoin-associated IBD reports in FAERS. Collectively, these findings indicate a period of litigation-driven overreporting likely occurred after 2009, and the exceptionally strong IBD signal detected here may largely reflect reporting bias.

Importantly, accumulating high-quality clinical evidence from recent years has consistently failed to demonstrate an independent association between isotretinoin exposure and IBD development. Large-scale population-based studies conducted over the past decade, along with updated meta-analyses and contemporary clinical guidelines, consistently suggest that IBD is predominantly driven by genetic susceptibility, environmental exposures, and immune dysregulation. These studies emphasize that such confounding factors frequently affect these patient populations, and that a direct causal relationship between isotretinoin and IBD has not been established [8,26–29]. Notably, large cohort studies employing propensity score–matched designs have shown that isotretinoin treatment, compared with appropriate acne-control populations, does not increase the risk of incident IBD [25,29]. Furthermore, several studies have reported that acne itself, rather than isotretinoin, is more strongly associated with subsequent IBD development, highlighting the critical role of confounding by indication in earlier observational findings. Systematic reviews and meta-analyses incorporating recent cohort data and trial sequential analysis further confirm no statistically significant association between isotretinoin use and IBD [26].

Collectively, although an exceptionally strong statistical signal for IBD was observed in this study, its clinical and pharmacological implications must be interpreted cautiously. Clinicians and regulatory authorities should remain attentive to shifts in reporting patterns, continuously integrate emerging high-quality clinical evidence, and provide scientifically grounded clarifications in drug labeling as appropriate. Such measures are essential for informed clinical decision-making and accurate public understanding of AE signals identified through pharmacovigilance systems.

#### 3.4.3. Teratogenicity of isotretinoin.

Isotretinoin can cross the placental barrier and is classified as pregnancy safety Grade X by the FDA. Clinical studies indicate that oral isotretinoin increases the risk of severe congenital malformations, spontaneous abortion, and premature birt [30]. This study found that isotretinoin ranked third among positive signals involving congenital, familial, and genetic disorders, mainly affecting the ears and labyrinth, central nervous system, cardiovascular system, musculoskeletal system, and eyes. This finding aligns with the prescribing information and existing literature [31,32]. The top ten congenital abnormalities identified were congenital middle ear abnormalities, anorectal malformations, congenital cerebellar hypoplasia, congenital optic nerve abnormalities, hemihypertrophy, congenital inner ear abnormalities, cerebellar dysplasia, anophthalmia, congenital ear malformations, and follicular keratosis. Special attention should be given to hemihypertrophy and follicular keratosis, which exhibited strong positive signals but are not listed in isotretinoin prescribing information. Current studies suggest potential associations between isotretinoin exposure and partial developmental asymmetry, unilateral abnormal growth, skin dysplasia, and keratinization disorders [33,34]. However, definitive correlations remain unconfirmed, and further research is necessary.

#### 3.4.4. Isotretinoin and pregnancy events.

This study identified 6,453 AEs related to pregnancy, puerperium, and perinatal conditions, of which 4,641 (71.92%) were specifically pregnancy-related. Among these, unexpected pregnancy demonstrated strong positive signals, ranking within the top 30 AE signals. These findings highlight the necessity for enhanced management of pregnancy events in clinical practice. Isotretinoin carries a high risk of severe birth defects and is contraindicated for pregnant women or those who may soon become pregnant. According to prescribing information in some countries, such as China, women of childbearing age or their spouses must use effective contraception from three months before isotretinoin initiation, during treatment, and for three months following cessation. The US FDA guidelines require contraception for at least one month before treatment, during treatment, and for three months after discontinuation.

Given the unequivocal teratogenic risk of isotretinoin, numerous pregnancy prevention programs (PPPs) have been implemented worldwide. Among these, the U.S. iPLEDGE program is widely considered one of the most stringent regulatory strategies for managing risk [35,36]. Comparative studies have demonstrated substantial heterogeneity among PPPs internationally regarding regulatory rigor, contraceptive requirements, frequency of pregnancy testing, and enforcement mechanisms. In particular, iPLEDGE imposes a significantly greater administrative burden on both patients and healthcare providers [37].

Despite these measures, real-world evidence indicates suboptimal effectiveness of PPPs. Although such programs have existed for many years, approximately 3.3%–6.5% of isotretinoin users still experience pregnancy during treatment [38]. Contributing factors likely include inadequate adherence to contraceptive regimens, insufficient patient education on pregnancy prevention, and inappropriate selection of contraception methods. Population-based studies and registry analyses from the United States and Europe further indicate that, in routine clinical practice, adherence to key PPP components, such as contraceptive use, regular pregnancy testing, and enrollment procedures, is frequently poor, with complete compliance rates often below 50% [37,39–41]. Moreover, previous studies has reported that some patients fail to fully recognize the profound teratogenic risks associated with isotretinoin before starting treatment. This lack of understanding, combined with poor contraceptive adherence, contributes to persistently high unintended pregnancy rates [38].

The limited success of PPPs is multifactorial. Patient-level factors include inadequate health literacy, limited access to contraceptive resources, and socioeconomic barriers. System-level challenges involve procedural complexity, pharmacy accessibility, and difficulties navigating digital platforms. Additionally, provider-level issues such as workflow burdens and inconsistent counseling quality further undermine effectiveness [39–42]. Recent real-world cohort studies also show notably lower adherence among younger women and socioeconomically disadvantaged groups [40,42]. Collectively, these findings indicate that while programs like iPLEDGE have significantly reduced fetal exposure risks, they remain insufficient to completely prevent pregnancies. This highlights the necessity for ongoing optimization of pregnancy prevention strategies informed by real-world adherence data.

Based on these findings, clinicians and pharmacists must consistently remind patients to rigorously use contraception during and after isotretinoin treatment. Patients should prioritize highly effective contraceptive methods or employ multiple contraceptive strategies. Pregnancy monitoring should be intensified, and strengthening patient education is critical for improving compliance. Regulatory agencies should further enhance and reinforce pregnancy prevention measures to ensure isotretinoin is used safely.

#### 3.4.5. Biological plausibility and context of novel AE signals.

Several AE signals identified in this study are not currently included in the product labeling for isotretinoin. Among the highest-ranked signals were nasal vestibulitis, anal papillary hypertrophy, neonatal neuroblastoma, diverticular hernia, and SAPHO syndrome.

From a mechanistic perspective, isotretinoin exerts therapeutic effects by modulating the retinoic acid signaling pathway. This modulation suppresses sebaceous gland activity and alters epithelial differentiation. Such effects are known to cause pronounced drying and thinning of the nasal mucosa [43], representing a plausible mechanism for isotretinoin-associated nasal vestibulitis. Clinical studies have demonstrated that systemic isotretinoin therapy can impair nasal mucociliary clearance and disrupt mucosal barrier integrity. These disruptions may increase susceptibility to microfissures and secondary bacterial colonization in the nasal vestibule [43, 44]. Although nasal vestibulitis is infrequently described as a discrete clinical endpoint, commonly reported treatment-related symptoms, including nasal dryness, irritation, and epistaxis, indirectly support inflammatory involvement of the nasal vestibule [43,44].

The biological plausibility of anal papillary hypertrophy may similarly reflect established regulatory effects of retinoic acid signaling on gastrointestinal epithelial proliferation and mucosal homeostasis [45, 46]. Experimental studies suggest that retinoic acid can exert trophic and remodeling effects on intestinal epithelium under specific conditions [45, 47]. Clinically, direct evidence linking isotretinoin to anal papillary hypertrophy remains limited. However, isotretinoin-induced mucosal dryness and altered epithelial turnover may plausibly increase susceptibility to local irritation and reactive hyperplasia.

Regarding neonatal neuroblastoma, biological considerations require careful interpretation. Neuroblastoma is an embryonal tumor originating from neural crest cells. Migration, differentiation, and survival of these cells during early embryogenesis depend heavily on tightly regulated retinoic acid signaling [48]. Experimental data suggest that excessive or dysregulated retinoic acid exposure during critical developmental periods may disrupt neural crest–related gene expression and apoptotic pathways. This disruption could theoretically predispose to developmental abnormalities [49]. However, current clinical and epidemiological evidence does not support a causal relationship between maternal isotretinoin exposure and neonatal neuroblastoma. Conversely, isotretinoin and other retinoids are widely employed as differentiation agents in treating high-risk neuroblastoma. Their antitumor activity primarily occurs through promoting cellular maturation and apoptosis rather than inducing tumorigenesis [50, 51]. Reports of isotretinoin embryopathy accompanied by congenital neuroblastoma exist only as isolated cases and are insufficient to establish causality [52].

The potential biological mechanism underlying diverticular hernia may involve the regulatory effects of retinoic acid signaling on extracellular matrix remodeling and collagen metabolism. These processes are critical for maintaining the structural integrity of the gastrointestinal wall [53]. Abnormalities in collagen composition and matrix turnover have been implicated in the development of diverticular disease and hernia formation [54].

SAPHO syndrome is currently not listed among adverse reactions in approved isotretinoin product labeling. Biologically, SAPHO syndrome is a rare autoinflammatory disorder characterized by dysregulated innate immune responses and abnormal bone remodeling. Retinoic acid signaling has well-established immunomodulatory effects and influences bone metabolism, including regulation of proinflammatory cytokines and osteogenic pathways. This provides mechanistic plausibility for retinoid-mediated modulation of SAPHO-related pathways in susceptible individuals [55–57]. Clinically, associations between isotretinoin and SAPHO syndrome have been primarily reported in sporadic case reports without systematic controlled studies. Notably, retinoids have also been used therapeutically in selected SAPHO syndrome patients, further complicating causal interpretation [58].

Overall, this study identified several previously unlabelled AE signals associated with isotretinoin that exhibited relatively strong disproportionality signals. However, the absolute number of reports for these events was generally small, potentially limiting the precision and robustness of the findings. Although biological plausibility exists for some associations, current clinical and epidemiological evidence remains insufficient to establish causality. Continued surveillance of pharmacovigilance databases, alongside well-designed clinical and mechanistic studies, is necessary to validate these signals further. The present findings provide preliminary guidance on safety issues warranting attention in clinical practice and future research.

#### 3.4.6. Analysis of external validation.

From an epidemiological baseline perspective, the EV and FAERS databases were highly comparable in background patient numbers, target drug report numbers, and patient sex and age distributions. These similarities strengthen the credibility of our findings. At the System Organ Class (SOC) level, results from the EV database were highly consistent with those from FAERS.

Specifically, three key aspects of concordance were observed. First, SOCs involved in overall AE reports and positive AE signals were identical between the two databases, confirming that isotretinoin-associated AEs affect multiple organ systems. Second, SOCs highly ranked in terms of signal counts and report frequencies in the FAERS database similarly ranked near the top in the EV database, with only minor ordering differences. In the EV database, psychiatric disorders, congenital, familial, and genetic disorders, and gastrointestinal disorders remained the top three SOCs ranked by signal count, confirming that AEs in these categories are among the most frequently encountered in clinical practice. Third, SOCs exhibiting cumulative positive signals in FAERS were also positive in the EV database. Notably, the EV database demonstrated an additional cumulative positive signal for congenital, familial, and genetic disorders. Further analysis revealed that this SOC was borderline positive in FAERS under the MGPS method, with an EBGM05 value of 1.99 (threshold >2), while the remaining three methods indicated positive signals.

Collectively, these findings further support the feasibility of the methodology and the robustness of the results. In the FAERS database, 469 positive AE signals associated with isotretinoin were identified, of which 396 (84.43%) overlapped with signals in the EV database. Among the top 30 signals, only Kleine–Levin syndrome and hemihypertrophy were not identified as positive in EV. This discrepancy likely results from the limited number of related reports in EV.

In addition, given the strong signal for inflammatory bowel disease (IBD) identified in EV, further analyses were performed using raw data. A total of 6,300 IBD reports were identified, with 6,112 submitted by non–healthcare professionals. Between 2009 and 2014, 5,547 IBD reports (88.05% of the total) were recorded. Since 2020, fewer than 10 IBD reports have been submitted annually. This temporal reporting pattern closely mirrors observations from FAERS, suggesting increased global reporting during a specific period. This spike may be attributed to heightened international awareness following events involving U.S. manufacturers.

Overall, the substantial overlap in positive signals between the two databases provides strong external validation, confirming the reliability and reproducibility of this study’s findings.

### 3.5. Clinical implications and integration of signal-mining findings into practice

Although disproportionality-based signal mining cannot establish causality at the individual patient level, it plays a critical role in contemporary pharmacovigilance by providing early warnings and prioritizing safety issues for further evaluation. The findings of this study may be integrated into clinical practice and pharmacovigilance systems in several ways.

First, clinicians may use high-ranking and reproducible signals identified across independent databases (FAERS and EudraVigilance) as triggers for enhanced clinical vigilance. For example, signals related to psychiatric disorders, pregnancy-related events, and rare but severe outcomes may prompt closer patient counseling, baseline risk assessment, and targeted follow-up during isotretinoin therapy, particularly in vulnerable populations.

Second, at the institutional level, hospital pharmacists and pharmacovigilance teams may incorporate such signal-mining results into local drug safety monitoring programs. Signals with high disproportionality and consistent external validation can be prioritized for periodic safety reviews, internal alerts, or integration into clinical decision support systems, thereby facilitating earlier detection of emerging safety concerns [59].

Finally, from a regulatory and systems perspective, signal-mining studies based on large spontaneous reporting databases support structured signal management processes, including signal validation, prioritization, and risk communication. When interpreted alongside clinical evidence and mechanistic plausibility, these data contribute to informed risk–benefit assessments and may guide updates to risk minimization measures or educational materials, as recommended in established pharmacovigilance frameworks [60].

## 4. Research Innovations and Limitations

### 4.1. Research innovations

#### 4.1.1. Methodological novelty and external validation.

We pioneered the systematic integration of four internationally recognized pharmacovigilance algorithms (ROR, PRR, BCPNN, MGPS). This integrated approach was applied comprehensively to 142,160 AE reports associated with isotretinoin over two decades (2004–2024) from the FAERS database. This approach significantly enhanced the robustness of signal detection and improved identification of rare events.

A key innovation was the inclusion of external validation using an independent pharmacovigilance database. AE signals identified for isotretinoin in FAERS were systematically replicated in the EudraVigilance database. The results showed high concordance in epidemiological characteristics, System Organ Class distributions, and signal detection outcomes. This dual-database framework enhances reproducibility, reduces database-specific bias, and strengthens the robustness and generalizability of the findings, supporting more reliable post-marketing safety assessments.

#### 4.1.2. Expansion of the risk profile.

We established the most comprehensive AE profile for isotretinoin to date, identifying 469 consolidated safety signals. Notably, 8 previously unlisted signals (including nasal vestibulitis, anal papilla hypertrophy, and neonatal neuroblastoma) provide preliminary insights into safety concerns that warrant attention in clinical practice and future research.

#### 4.1.3. Novel risk management findings.

Unintended pregnancy (ROR = 91.39) emerged as the strongest pregnancy-related signal. This finding provides critical empirical evidence for optimizing global Pregnancy Prevention Programs (e.g., iPLEDGE) and highlights potential vulnerabilities in current risk management protocols.

### 4.2. Research limitations

Although this study analyzed extensive real-world data, certain limitations remain. (1) The analyzed data primarily originated from European and American countries, which might be influenced by racial and regional differences. (2) Due to the spontaneous nature of reporting databases, information might be misreported or underreported, potentially affecting the accuracy of results. (3) Because the study is based on spontaneous reporting data, detected AE signals only indicate statistical associations rather than causality. High signal strength does not necessarily reflect high clinical risk due to potential reporting bias and confounding factors. These signals thus require validation through prospective studies, mechanistic research, or clinical trials. This study also cannot calculate or infer incidence, relative risk, or absolute risk of AEs. The FAERS database lacks reliable information on the number of exposed patients (denominator). Reporting behavior is influenced significantly by non-pharmacological factors such as medical practices, public awareness, media attention, and legal activities. This leads to substantial underreporting and reporting bias. Consequently, signal strength (e.g., ROR value) reflects statistical disproportionality, not the actual frequency or magnitude of risk. Signal mining serves to identify potential safety issues warranting further rigorous investigation (e.g., prospective cohort studies).

## 5. Conclusions

In summary, this study utilized the FAERS database and employed SAS 9.4 software with four signal detection methods to identify AE signals associated with isotretinoin in real-world settings. Findings indicated that AEs were primarily concentrated in psychiatric and gastrointestinal disorders, aligning with isotretinoin’s prescribing information. Clinical monitoring and follow-up should therefore be strengthened. Notably, although inflammatory bowel disease (IBD) exhibited a strong statistical signal, reports were predominantly submitted by legal professionals from 2009 to 2014, indicating a potential litigation-driven reporting bias. Accordingly, interpretation of the IBD signal requires caution, as its magnitude may be substantially overestimated. Moreover, current high-quality clinical evidence supports that isotretinoin should not be considered an independent causal factor for IBD. The study also identified AE signals not currently documented in prescribing information. These included nasal vestibulitis, hypertrophic anal papilla, neonatal neuroblastoma, diverticular hernia, SAPHO syndrome, somatic delusional disorder, hypersomnia-bulimia syndrome, and hemihypertrophy. While some signals have plausible pharmacological mechanisms, their rarity and lack of causal evidence suggest their main value lies in hypothesis generation for future mechanistic studies and epidemiological surveillance, rather than immediate clinical decisions. In addition, strong signals related to severe congenital malformations and pregnancy-related events highlight the continued necessity for enhanced patient education, strict contraceptive counseling, and enhanced pregnancy prevention and management strategies to ensure isotretinoin’s safe use. External validation using the European EudraVigilance database demonstrated high concordance with FAERS regarding epidemiological characteristics, System Organ Class distributions, and signal detection outcomes. This dual-database validation framework reduces single-database bias, enhances robustness and generalizability, and provides more reliable evidence for post-marketing drug safety assessments. Nevertheless, these identified signals represent statistical associations rather than causation. Clinical interpretation should be individualized and cautious, with appropriate enhanced monitoring. Further high-quality clinical and epidemiological studies are necessary to clarify the clinical relevance and causal nature of these signals.

## Supporting information

S1 Table Statistical Analysis of PT Signals for Isotretinoin Adverse Events in FAERS database. (XLSX)

S2 Table Statistical Analysis of SOC Involved in Adverse Event Signals of Isotretinoin in FAERS database. (XLSX)

S3 Table Statistical Analysis of PT Signals for Isotretinoin Adverse Events in EV database. (XLSX)

S4 Table Statistical Analysis of SOC Involved in Adverse Event Signals of Isotretinoin in EV database. (XLSX)

S5 Table Adverse Events from Top 30 Signals Not Documented in the Label in FAERS database. (XLSX)
