# Supplementary material for: Signal mining and analysis of adverse events associated with Isotretinoin: A 20-Year real-world pharmacovigilance study based on FAERS and EudraVigilance databases
Source: PLoS One. 2026 Jul 16;21(7):e0329161. doi: 10.1371/journal.pone.0329161 (PMC13483711; doi:10.1371/journal.pone.0329161)
Supplement: Supplementary file 1 [file pone.0329161.s006.docx]

Peer Review Report

Title of the manuscript:

**Signal Mining and Analysis of Adverse Events of Isotretinoin: 20-year real-world pharmacovigilance analysis based on the FAERS database**

General Assessment

This manuscript addresses a highly relevant and timely topic in dermatology. Isotretinoin remains a cornerstone in the treatment of severe acne, yet its safety profile, particularly concerning psychiatric disorders, inflammatory bowel disease (IBD), and teratogenicity, continues to be the subject of debate.

The authors employ a robust methodological approach by integrating four pharmacovigilance signal detection methods (ROR, PRR, BCPNN, and MGPS) to analyze adverse events (AEs) associated with isotretinoin based on 20 years of data from the FAERS database.

The study contributes to the field by consolidating known risks and identifying eight potential new AE signals not currently included in the drug label. However, **despite its merits, the manuscript requires major revisions prior to acceptance.**

**Major Comments**

Causality vs. Association

The study relies on spontaneous reports, which inherently reflect association rather than causality. This critical distinction must be emphasized throughout the discussion and conclusion sections to avoid overinterpretation of signal strength.

Bias in IBD Reports

The authors acknowledge that over 84% of IBD reports were submitted by lawyers between 2009 and 2014, suggesting significant legal-driven reporting bias. While this is an important observation, the current framing may still overstate the strength of this signal. A more cautious interpretation is warranted.

New AE Signals

The inclusion of signals such as neonatal neuroblastoma, hypertrophic anal papilla, and hemihypertrophy is intriguing. However, these findings require more context regarding biological plausibility, previous literature, or mechanistic hypotheses before being suggested as candidates for label revision.

Reference Errors

Multiple entries throughout the manuscript display the placeholder “Error! Reference source not found.” This detracts from scientific credibility and should be resolved before further review.

Methodological Clarity

While the statistical methodology is robust, the threshold definitions (Table 2) are overly technical. The authors should include brief plain-language explanations to improve accessibility to a broader audience.

Discussion of Teratogenicity and Pregnancy Events

The paper successfully highlights the persistent issue of unintended pregnancies during isotretinoin use. The discussion would benefit from a comparative overview of pregnancy prevention programs (e.g., iPLEDGE) and why such systems may fail, including real-world patient compliance data where available.

Minor Comments

Please clarify whether the data extraction from FAERS was fully automated or manually curated after de-duplication.

In the “Limitations” section, explicitly state that incidence cannot be inferred from FAERS data.

It would be useful to include a supplemental table summarizing the eight novel AE signals not previously included in the label, with frequencies and reporting years.

Recommendation

**Major Revision**

This is a comprehensive and potentially impactful pharmacovigilance study. However, improvements in the interpretation of key signals, discussion of biological plausibility, correction of formatting issues, and refinement of conclusions are necessary prior to publication.
