# Supplementary material for: Signal mining and analysis of adverse events associated with Isotretinoin: A 20-Year real-world pharmacovigilance study based on FAERS and EudraVigilance databases
Source: PLoS One. 2026 Jul 16;21(7):e0329161. doi: 10.1371/journal.pone.0329161 (PMC13483711; doi:10.1371/journal.pone.0329161)
Supplement: Supplementary file 2 [file pone.0329161.s008.docx]

**Responses to Academic Editor and Reviewers**

**Section 1: Response Letter............................................................................................1**

**Section 2: Responses to Academic Editor ....................................................................3**

**Section 3: Responses to reviewer# 1............................................................................7**

***Note:***

- *For your convenience, clickable links have been embedded in the table of contents above. You can press* ***Ctrl + Left-click on the blue section title*** *to jump directly to the corresponding part of the text.*
- *The page and line numbers of the revisions marked in this response letter correspond to the following document:*
- *PDF version of the document titled "Revised Manuscript with Track Changes"*

**Section 4: Responses to reviewer# 2 ..........................................................................22**

# Section 1：Response Letter

**Dear Editor and Reviewers,**

First and foremost, we would like to extend our sincere gratitude to you for your time and effort in reviewing our manuscript and providing us with such valuable, insightful, and constructive comments. Your feedback is of immense importance, offering crucial guidance not only for the revision and improvement of our current manuscript but also for inspiring our future research endeavors.

We have placed the highest priority on all the suggestions received. Upon receiving the review comments, our team engaged in thorough discussions and undertook a comprehensive and meticulous revision of the manuscript. We have addressed each point raised, leading to systematic enhancements in the methodological description, interpretation of results, depth of discussion, rigor of conclusions, as well as the language and formatting throughout the text. All modifications have been highlighted in color in the revised manuscript, and our detailed considerations along with specific locations of changes are provided in the point-by-point responses below.

It is important to note that we are fully aware of the current high standards in academic publishing, especially for studies based on real-world data mining, which demand robust and reproducible results. Therefore, despite the significant increase in data processing and analytical workload, **we were committed to conducting a systematic external validation using the European EudraVigilance database**. While not explicitly requested by the reviewers, we believe that demonstrating consistent findings across two independent, large-scale pharmacovigilance databases significantly strengthens the credibility and academic value of our conclusions. This additional work, however, substantially increased the complexity and time required for the revision process.

Our major revisions, undertaken in direct response to your key concerns, include the following:

1. **Enhanced Methodological Transparency and Result Interpretation:** We have added detailed descriptions of the data processing workflow and provided plain-language explanations of key statistical thresholds to improve readability and reproducibility.
2. **Deepened Discussion and Cautious Interpretation:** We have significantly strengthened the critical discussion of key signals (e.g., Inflammatory Bowel Disease), delving into potential reporting biases and integrating the most recent high-quality clinical evidence for balanced interpretation. A dedicated discussion on the biological plausibility of novel signals has been added.
3. **Incorporation of External Validation for Robustness:** We introduced a parallel analysis based on the EudraVigilance database, using cross-database consistency comparisons to further verify the reliability of our core findings.
4. **Improved Formatting and Referencing:** We have carefully checked and corrected all formatting issues per the journal's requirements, ensuring complete and accurate references, proper in-text citations for all tables and figures, and providing full captions for all Supporting Information files.
5. **Expanded Clinical and Regulatory Implications:** We added a new subsection discussing how signal-mining findings can be integrated into clinical practice and pharmacovigilance systems to enhance the real-world impact of the study.
6. **Comprehensive Language Polishing:** The entire manuscript has undergone professional English language editing to optimize clarity, fluency, and academic consistency.

At this point, we feel it is necessary to offer our most sincere apologies and provide a candid explanation for the delay in submitting this revision. The revision process was considerably challenged by the complexity of data processing and analysis, particularly due to the newly added external validation component. Compounding this, our team faced an unexpected personal hardship: the first author, who was primarily responsible for the core data analysis, experienced severe health challenges during this period due to a high-risk pregnancy, accompanied by exceptionally difficult physical reactions that necessitated periods of bed rest. Deeply committed to this research, she endeavored to resume work as soon as her condition slightly improved, striving to contribute to the revisions despite the circumstances. The confluence of these factors severely impacted our overall progress, leading to the regrettable delay. We fully understand and respect the timelines inherent to academic publishing and deeply regret any inconvenience this may have caused. We sincerely hope for your understanding regarding this exceptional situation. Despite the difficulties, we remained steadfast in our commitment to scientific rigor, valuing this critical revision opportunity immensely. We were determined to submit a manuscript that withstands scrutiny, refusing to compromise on the depth or quality of revisions due to time constraints.

We firmly believe that incorporating your expert insights and undertaking these additional efforts have significantly enhanced the scientific rigor, logical clarity, and academic value of the manuscript. Once again, we express our heartfelt thanks for your dedication, expert guidance, and kind understanding. We look forward to your further review and are prepared to fully cooperate with any subsequent modification requests.

**Sincerely,**

**Yuan Bian**

# Section 2：Responses to Academic Editor

**(original comments by Editor are in blue color)**

1. **Comment:**

Please ensure that your manuscript meets PLOS ONE's style requirements, including those for file naming. The PLOS ONE style templates can be found at

https://journals.plos.org/plosone/s/file?id=wjVg/PLOSOne_formatting_sample_main_body.pdf and <https://journals.plos.org/plosone/s/file?id=ba62/PLOSOne_formatting_sample_title_authors_affiliations.pdf>

**Reply:**

Dear Editor,

Thank you very much for pointing this out. We fully understand and place high importance on adhering to the specific style requirements of PLOS ONE to ensure the smooth processing of our manuscript through the publication pipeline.

We have carefully reviewed the PLOS ONE style template links you provided and will strictly implement all relevant specifications in the revised manuscript. Our specific actions will include:

1.File Naming: We will ensure that all submitted files (main manuscript, figures, supplementary materials, etc.) follow the required naming conventions as per PLOS ONE's submission guidelines.

2.Template Application and Comprehensive Check: We will reformat our manuscript using the official templates (particularly the title/authors/affiliations template) as the baseline. This will encompass, but not be limited to:

- Adjusting the title page layout, author affiliation listing, and corresponding author designations.
- Standardizing the hierarchy and font formatting of section headings within the main text.
- Ensuring all figures, tables, and their legends comply with PLOS ONE's labeling and submission requirements.
- Converting both the in-text citation style and the end-of-text reference list format to the Vancouver style as required by PLOS ONE.

We confirm that these changes pertain solely to formatting and style and will not alter the scientific content, data, or conclusions of the paper. We appreciate your guidance in ensuring the manuscript meets the journal's standards.

2. **Comment:**

We are unable to open your Supporting Information file “Supporting Information.zip”. Please kindly revise as necessary and re-upload.

**Reply:**

Dear Editor,

Thank you for your note regarding the Supporting Information file. We apologize for the technical issue with the “Supporting Information.zip” archive and appreciate the opportunity to rectify it.

In response, we have thoroughly revised the Supporting Information in accordance with PLOS formatting guidelines. Specifically:

1. We have replaced the compressed archive with four individually named XLSX files (S1_Table.xlsx, S2_Table.xlsx, S3_Table.xlsx, S4_Table.xlsx，S5_Table.xlsx), each corresponding to the supporting tables referenced in the manuscript.
2. Each file is under 10 MB in size and contains complete statistical outputs from our disproportionality analyses in both the FAERS and EV databases.
3. We have updated the “Supporting Information” section in the manuscript.

These changes ensure that all supporting data are directly accessible without dependency on compression software, thereby aligning with PLOS recommendations for supporting information delivery.

The revised files have been uploaded to the submission system. Should any further adjustments be needed, we are happy to comply.Thank you for your guidance and support.

3. **Comment:**

We note you have included a table to which you do not refer in the text of your manuscript. Please ensure that you refer to Table 5 in your text; if accepted, production will need this reference to link the reader to the Table.

**Reply:**

Dear Editor,

Thank you for your careful review and constructive comment. We have noted the omission of the citation for Table 5 in the manuscript text. As suggested, we have now inserted a clear reference to Table 5 in Section 5.3 (“Classification of system organs involved in AE signals related to isotretinoin in the FAERS database”) where the SOC distribution and cumulative signal intensities are discussed.

Specifically, we revised the sentence to read:“Signal intensities for SOC-related AEs associated with isotretinoin are presented in Table 5.”

Furthermore, we have conducted a comprehensive check of all figure, table, and supporting information citations throughout the manuscript. We confirm that all other references (to Figures 1-3, Tables 1-4, 6-8, and S1-S4 Tables) are appropriately placed and meet the journal's formatting requirements.

This revision ensures that Table 5 is properly referenced, and our full self-audit guarantees overall consistency in citation, facilitating reader navigation and fully complying with journal production standards.

We appreciate your attention to detail and hope the manuscript now meets all required criteria.

- **Please see below for the details: （Page 12 , Line 477-478，the section highlighted in blue）**

Signal intensities for SOC-related AEs associated with isotretinoin are presented in Table 5.

4. **Comment:**

Please include captions for your Supporting Information files at the end of your manuscript, and update any in-text citations to match accordingly. Please see our Supporting Information guidelines for more information: http://journals.plos.org/plosone/s/supporting-information.

**Reply:**

Dear Editor,

Thank you for your valuable feedback regarding the captions for the Supporting Information files and the corresponding in-text citations. We greatly appreciate your clear guidance and have now duly revised the manuscript accordingly.

Specifically, we have:

1.Added complete captions for all Supporting Information files at the end of the manuscript, following PLOS formatting guidelines;

2.Updated the relevant in-text citations throughout the manuscript to ensure consistency.

These additions further improve the transparency and accessibility of the supporting data, helping readers better understand the relationship between the key findings presented in the text and the complete datasets provided.

The revised manuscript, which now includes the full Supporting Information captions and updated citations, has been re-uploaded to the submission system.

We believe these modifications fully address the points you raised. Should any further adjustments be needed, we are pleased to cooperate.

Thank you again for your review and support.

- **Please see below for the details:**

1. Specific details are shown in S1 and S5 Table.（Page 10 , Line 456-457，the section highlighted in blue）
2. Specific details are shown in S2 Table.（Page 12, Line 474-475，the section highlighted in blue）
3. Specific details are shown in S3 Table.（Page 17, Line 644-645，the section highlighted in blue）
4. Specific details are shown in S4 Table.（Page 18 , Line 661-662，the section highlighted in blue）
5. Supporing information（Page 41-42 , Line 1889-1898，the section highlighted in blue）

S1 Table. Statistical Analysis of PT Signals for Isotretinoin Adverse Events in FAERS database.

S2 Table. Statistical Analysis of SOC Involved in Adverse Event Signals of Isotretinoin in FAERS database.

S3 Table. Statistical Analysis of PT Signals for Isotretinoin Adverse Events in EV database.

S4 Table. Statistical Analysis of SOC Involved in Adverse Event Signals of Isotretinoin in EV database.

S5_Table.Adverse Events from Top 30 Signals Not Documented in the Label in FAERS database

5. **Comment:**

If the reviewer comments include a recommendation to cite specific previously published works, please review and evaluate these publications to determine whether they are relevant and should be cited. There is no requirement to cite these works unless the editor has indicated otherwise.

**Reply:**

Dear Editor,

Thank you for your valuable feedback. We fully acknowledge your note regarding the citation of previously published works. In this revision, we have carefully evaluated all references added during the modification process. Each citation was included solely based on its direct relevance to the methodological rigor, contextual background, or interpretive framework of our study. No citation was added due to external requests; rather, all references were selected to strengthen the scholarly foundation and transparency of our work. The added citations are appropriately placed in the Introduction, Methods, and Discussion sections as needed. We believe these additions enhance the academic integrity and clarity of the manuscript without unnecessary redundancy.

Thank you again for your review and support.

# Section 3: Responses to reviewer# 1

**(original comments are in green color)**

1. **Comment:**

**Major Comments**

Causality vs. Association

The study relies on spontaneous reports, which inherently reflect association rather than causality. This critical distinction must be emphasized throughout the discussion and conclusion sections to avoid overinterpretation of signal strength.

**Reply:**

Dear Reviewer,

Thank you for your thorough review and valuable comments on our manuscript. You rightly pointed out that spontaneous reporting data reflect association rather than causality, which is a crucial distinction for enhancing the scientific rigor of this paper. We fully agree with your comment and have revised the Discussion and Conclusion sections accordingly to clarify the difference between “association” and “causality” and to avoid overinterpretation of signal strength.

Specifically, we have made the following revisions:

1. Added a paragraph at the beginning of the Discussion section to emphasize the associative nature of spontaneous reporting data.

- **Please see below for the details: （Page 20 , Line 736-755，the section highlighted in green）**

This study systematically analyzed AE signals related to isotretinoin using the FAERS spontaneous reporting system and four statistical methods. **It is important to note that spontaneous reporting data only indicate statistical associations between drugs and** AE**s, not causality.** Signal detection aims to identify potential risk signals to guide future clinical research, regulatory decisions, and clinical monitoring, rather than confirming direct causation. Therefore, the following discussion should be framed within the context of “association,” avoiding overinterpretation.

2. Incorporated cautious language in each key signal discussion subsection, explicitly stating that signals represent statistical associations only.

- **Please see below for the details: （Page 23, Line 950-953，the section highlighted in green）**

It should be noted that psychiatric signals detected in this study represent statistical associations only and do not imply direct causation by isotretinoin. Acne itself and its associated psychosocial stress may serve as confounding factors for psychiatric symptoms. Future prospective cohort or case-control studies are required to further investigate causality.

3. Further elaborated on the interpretative limitations due to data nature in the Study Limitations section.

- **Please see below for the details: （Page 34, Line 1541-1545，the section highlighted in green）**

(3) Because the study is based on spontaneous reporting data, detected AE signals only indicate statistical associations rather than causality. High signal strength does not necessarily reflect high clinical risk due to potential reporting bias and confounding factors. These signals thus require validation through prospective studies, mechanistic research, or clinical trials.

4. Clearly stated in the Conclusion that the findings are “associative signals” requiring further validation.

- **Please see below for the details: （Page 35 , Line 1627-1630，the section highlighted in green）**

Nevertheless, these identified signals represent statistical associations rather than causation. Clinical interpretation should be individualized and cautious, with appropriate enhanced monitoring. Further high-quality clinical and epidemiological studies are necessary to clarify the clinical relevance and causal nature of these signals.

These revisions help to present our findings and their implications more accurately, aligning with the scientific standards of pharmacovigilance research. We appreciate your constructive feedback.

2. **Comment:**

**Major Comments**

Bias in IBD Reports

The authors acknowledge that over 84% of IBD reports were submitted by lawyers between 2009 and 2014, suggesting significant legal-driven reporting bias. While this is an important observation, the current framing may still overstate the strength of this signal. A more cautious interpretation is warranted.

**Reply:**

Dear Reviewer,

We sincerely thank you for raising this insightful and critical point. We fully concur with your observation that “the IBD reports may be subject to legal-driven reporting bias, and the current framing might overstate the signal strength, warranting a more cautious interpretation.” This comment precisely addresses a core methodological challenge in pharmacovigilance research based on spontaneous reporting systems. Adopting your recommendation is crucial for enhancing the balance and scientific soundness of our conclusions.

Accordingly, we have implemented substantial revisions to the manuscript, primarily reflected in the following two aspects:

1. The “Discussion” section (3.4.2 Isotretinoin and IBD) has been thoroughly rewritten and expanded. We not only report that 84.94% of IBD reports were submitted by lawyers but further analyze their highly specific temporal pattern (85.75% of reports concentrated between 2009-2014, with annual reports plummeting to single digits after 2018). We link this pattern to the period of litigation activity against isotretinoin manufacturers in the US during the same timeframe, citing dedicated literature to substantiate this as a known phenomenon of “legal-driven over-reporting.” More importantly, we proactively integrate the latest high-quality evidence-based medical findings, including large-scale propensity score-matched cohort studies and the most recent meta-analysis incorporating trial sequential analysis. These studies consistently indicate that, after controlling for confounders (particularly the indication of “acne” itself), isotretinoin does not demonstrate an independent causal association with IBD. This revision situates the strong statistical signal identified in our study within the broader context of clinical evidence, clearly distinguishing between “statistical association” and “clinical causation.”

- **Please see below for the details: （Page 23-25, Line 965-1081，the section highlighted in green）**

**3.4.2 Isotretinoin and AE signals related to IBD**

This study identified an exceptionally strong signal for inflammatory bowel disease (IBD), with a reporting odds ratio (ROR) of 579.14, ranking first among all detected signals and second in the number of reported cases, indicating an unusually high statistical strength. Given the extraordinary magnitude of the IBD signal, a detailed analysis of reporting patterns was conducted to better contextualize this finding.

In total, 5,278 IBD reports were identified, of which 4,483 (84.94%) were submitted by legal professionals. Temporal analysis further revealed a significant clustering of reports between 2009 and 2014, during which 4,526 cases (85.75%) occurred. In contrast, since 2018, fewer than 10 IBD reports have been submitted annually. This markedly skewed distribution in reporter type and reporting period strongly suggests non-random reporting dynamics.

To further elucidate the potential reasons for the strong isotretinoin-IBD association observed, the broader regulatory and legal context was examined. Following 2009, multiple high-profile lawsuits were filed in the United States against isotretinoin manufacturers, coinciding with a marked surge in IBD reports from 2009–2012. Previous investigations indicated that a substantial proportion of FAERS reports linking isotretinoin to IBD originated from law firms, highlighting potential litigation-driven stimulated reporting [24-25]. The reporting patterns observed in the present study closely align with known timelines of litigation activity in the United States. These results are consistent with previous studies documenting attorney-driven waves of isotretinoin-associated IBD reports in FAERS. Collectively, these findings indicate a period of litigation-driven overreporting likely occurred after 2009, and the exceptionally strong IBD signal detected here may largely reflect reporting bias.

Importantly, accumulating high-quality clinical evidence from recent years has consistently failed to demonstrate an independent association between isotretinoin exposure and IBD development. Large-scale population-based studies conducted over the past decade, along with updated meta-analyses and contemporary clinical guidelines, consistently suggest that IBD is predominantly driven by genetic susceptibility, environmental exposures, and immune dysregulation. These studies emphasize that such confounding factors frequently affect these patient populations, and that a direct causal relationship between isotretinoin and IBD has not been established[8,26-29]. Notably, large cohort studies employing propensity score–matched designs have shown that isotretinoin treatment, compared with appropriate acne-control populations, does not increase the risk of incident IBD [25,29]. Furthermore, several studies have reported that acne itself, rather than isotretinoin, is more strongly associated with subsequent IBD development, highlighting the critical role of confounding by indication in earlier observational findings. Systematic reviews and meta-analyses incorporating recent cohort data and trial sequential analysis further confirm no statistically significant association between isotretinoin use and IBD [26].

Collectively, although an exceptionally strong statistical signal for IBD was observed in this study, its clinical and pharmacological implications must be interpreted cautiously. Clinicians and regulatory authorities should remain attentive to shifts in reporting patterns, continuously integrate emerging high-quality clinical evidence, and provide scientifically grounded clarifications in drug labeling as appropriate. Such measures are essential for informed clinical decision-making and accurate public understanding of AE signals identified through pharmacovigilance systems.

**References：**

24.Stobaugh DJ, Deepak P, Ehrenpreis ED. Alleged isotretinoin-associated inflammatory bowel disease: disproportionate reporting by attorneys to the food and drug administration adverse event reporting system. J Am Acad Dermatol. 2013;69: 393–398. doi:10.1016/j.jaad.2013.04.031

25.Tenner S. Isotretinoin and inflammatory bowel disease: trial lawyer misuse of science and FDA warnings. Am J Gastroenterol. 2014;109: 570–571. doi:10.1038/ajg.2014.34

26.Yu C-L, Chou P-Y, Liang C-S, Chiang L-H, Wang T-Y, Tu Y-K, et al. Isotretinoin exposure and risk of inflammatory bowel disease: a systematic review with meta-analysis and trial sequential analysis. Am J Clin Dermatol. 2023;24: 721–730. doi:10.1007/s40257-023-00765-9

27.Kridin K, Ludwig RJ. Isotretinoin and the risk of inflammatory bowel disease and irritable bowel syndrome: a large-scale global study. J Am Acad Dermatol. 2023;88: 824–830. doi:10.1016/j.jaad.2022.12.015

28.Popescu CM, Bigby M. The weight of evidence on the association of isotretinoin use and the development of inflammatory bowel disease. JAMA Dermatol. 2013;149: 221–222. doi:10.1001/jamadermatol.2013.1348

29.Taylor MT, Margolis DJ, Kwatra SG, Barbieri JS. A propensity score matched cohort study identifying an association of acne, but not oral antibiotic or isotretinoin use, with risk of incident inflammatory bowel disease. J Am Acad Dermatol. 2023;88: 841–847. doi:10.1016/j.jaad.2023.01.014

2. An explicit cautionary statement has been added to the “Conclusion” section. We specifically note that although IBD shows an exceptionally high statistical association signal, its interpretation must be contextualized by the evident legal-driven reporting bias, and current high-quality clinical evidence does not support regarding it as an independent causal risk factor. This ensures the final conclusion of the paper maintains the necessary caution.

- **Please see below for the details: （Page 35, Line 1609-1613，the section highlighted in green）**

Notably, although inflammatory bowel disease (IBD) exhibited a strong statistical signal, reports were predominantly submitted by legal professionals from 2009 to 2014, indicating a potential litigation-driven reporting bias. Accordingly, interpretation of the IBD signal requires caution, as its magnitude may be substantially overestimated. Moreover, current high-quality clinical evidence supports that isotretinoin should not be considered an independent causal factor for IBD.

Through these revisions, our discussion of the IBD signal has evolved from initially noting “potential reporting bias” to systematically arguing that “this signal is highly likely driven by reporting bias specific to a historical period and is contradicted by current mainstream clinical evidence.” We believe this fully addresses your call for “a more cautious interpretation,” significantly enhancing the scholarly rigor and reliability of the manuscript’s conclusions. We are grateful again for your insightful review, which has been instrumental in refining this study.

3. **Comment:**

**Major Comments**

New AE Signals

The inclusion of signals such as neonatal neuroblastoma, hypertrophic anal papilla, and hemihypertrophy is intriguing. However, these findings require more context regarding biological plausibility, previous literature, or mechanistic hypotheses before being suggested as candidates for label revision.

**Reply:**

Dear Reviewer,

We sincerely thank the reviewer for highlighting the need for greater context regarding the novel adverse event (AE) signals. We fully agree that discussing biological plausibility and existing literature is crucial before considering any implications for drug labeling. This constructive suggestion has significantly strengthened our manuscript.

In direct response to your comment, we have made the following key revisions:

We have added a dedicated subsection within the Discussion: “3.4.5 Biological Plausibility and Context of Novel AE Signals”. In this new section, we systematically address the signals of particular interest (e.g., neonatal neuroblastoma, hypertrophic anal papilla, nasal vestibulitis, SAPHO syndrome).

For each signal, we provide a concise review of potential biological mechanisms and reference existing literature:

For neonatal neuroblastoma, we discuss the critical role of retinoic acid signaling in neural crest cell development (providing biological plausibility) and cite literature on retinoid use in neuroblastoma therapy, while clearly stating the lack of epidemiological evidence for a causal link with isotretinoin exposure.

For hypertrophic anal papilla and nasal vestibulitis, we link them to isotretinoin’s well-known mucocutaneous effects on epithelial dryness and keratinization, suggesting a plausible pathway for localized tissue reaction.

For SAPHO syndrome, we discuss the immunomodulatory and osteometabolic effects of retinoids as a potential mechanistic link, while acknowledging the evidence is limited to sporadic case reports.

We have revised the framing in the Abstract and Conclusions. We now consistently describe these novel signals as “hypothesis-generating” findings that “highlight areas for future research and heightened clinical awareness” rather than suggesting immediate label revision. We emphasize the rarity of reports and the absence of established causality.

These revisions provide the necessary scientific context, enhance the depth of our discussion, and ensure a cautious, balanced interpretation of the novel signals—directly addressing your valuable feedback. We believe the manuscript is now substantially improved. Thank you again for your review and support.

- **Please see below for the details: （Page 28-30, Line 1258-1382，the section highlighted in green）**

**3.4.5 Biological plausibility and context of novel AE signals**

Several AE signals identified in this study are not currently included in the product labeling for isotretinoin. Among the highest-ranked signals were nasal vestibulitis, anal papillary hypertrophy, neonatal neuroblastoma, diverticular hernia, and SAPHO syndrome.

From a mechanistic perspective, isotretinoin exerts therapeutic effects by modulating the retinoic acid signaling pathway. This modulation suppresses sebaceous gland activity and alters epithelial differentiation. Such effects are known to cause pronounced drying and thinning of the nasal mucosa [43], representing a plausible mechanism for isotretinoin-associated nasal vestibulitis. Clinical studies have demonstrated that systemic isotretinoin therapy can impair nasal mucociliary clearance and disrupt mucosal barrier integrity. These disruptions may increase susceptibility to microfissures and secondary bacterial colonization in the nasal vestibule[43-44]. Although nasal vestibulitis is infrequently described as a discrete clinical endpoint, commonly reported treatment-related symptoms, including nasal dryness, irritation, and epistaxis, indirectly support inflammatory involvement of the nasal vestibule [43-44].

The biological plausibility of anal papillary hypertrophy may similarly reflect established regulatory effects of retinoic acid signaling on gastrointestinal epithelial proliferation and mucosal homeostasis [45-46]. Experimental studies suggest that retinoic acid can exert trophic and remodeling effects on intestinal epithelium under specific conditions [45,47]. Clinically, direct evidence linking isotretinoin to anal papillary hypertrophy remains limited. However, isotretinoin-induced mucosal dryness and altered epithelial turnover may plausibly increase susceptibility to local irritation and reactive hyperplasia.

Regarding neonatal neuroblastoma, biological considerations require careful interpretation. Neuroblastoma is an embryonal tumor originating from neural crest cells. Migration, differentiation, and survival of these cells during early embryogenesis depend heavily on tightly regulated retinoic acid signaling [48]. Experimental data suggest that excessive or dysregulated retinoic acid exposure during critical developmental periods may disrupt neural crest–related gene expression and apoptotic pathways. This disruption could theoretically predispose to developmental abnormalities[49]. However, current clinical and epidemiological evidence does not support a causal relationship between maternal isotretinoin exposure and neonatal neuroblastoma. Conversely, isotretinoin and other retinoids are widely employed as differentiation agents in treating high-risk neuroblastoma. Their antitumor activity primarily occurs through promoting cellular maturation and apoptosis rather than inducing tumorigenesis [50-51]. Reports of isotretinoin embryopathy accompanied by congenital neuroblastoma exist only as isolated cases and are insufficient to establish causality[52].

The potential biological mechanism underlying diverticular hernia may involve the regulatory effects of retinoic acid signaling on extracellular matrix remodeling and collagen metabolism. These processes are critical for maintaining the structural integrity of the gastrointestinal wall [53]. Abnormalities in collagen composition and matrix turnover have been implicated in the development of diverticular disease and hernia formation [54].

SAPHO syndrome is currently not listed among adverse reactions in approved isotretinoin product labeling. Biologically, SAPHO syndrome is a rare autoinflammatory disorder characterized by dysregulated innate immune responses and abnormal bone remodeling. Retinoic acid signaling has well-established immunomodulatory effects and influences bone metabolism, including regulation of proinflammatory cytokines and osteogenic pathways. This provides mechanistic plausibility for retinoid-mediated modulation of SAPHO-related pathways in susceptible individuals [55-57]. Clinically, associations between isotretinoin and SAPHO syndrome have been primarily reported in sporadic case reports without systematic controlled studies. Notably, retinoids have also been used therapeutically in selected SAPHO syndrome patients, further complicating causal interpretation [58].

Overall, this study identified several previously unlabelled AE signals associated with isotretinoin that exhibited relatively strong disproportionality signals. However, the absolute number of reports for these events was generally small, potentially limiting the precision and robustness of the findings. Although biological plausibility exists for some associations, current clinical and epidemiological evidence remains insufficient to establish causality. Continued surveillance of pharmacovigilance databases, alongside well-designed clinical and mechanistic studies, is necessary to validate these signals further. The present findings provide preliminary guidance on safety issues warranting attention in clinical practice and future research.

**References：**

43.Tasli H, Yurekli A, Gokgoz MC, Karakoc O. Effects of oral isotretinoin therapy on the nasal cavities. Braz J Otorhinolaryngol. 2020;86: 99–104. doi:10.1016/j.bjorl.2018.10.004

44.İşlek A, Yıldız MG. Evaluation of nasal mucociliary clearance, nasal obstruction symptom evaluation, and epistaxis severity score in isotretinoin treatment. Ear Nose Throat J. 2021;100: 566–569. doi:10.1177/0145561320920425

45.Wang L, Tang Y, Rubin DC, Levin MS. Chronically administered retinoic acid has trophic effects in the rat small intestine and promotes adaptation in a resection model of short bowel syndrome. Am J Physiol Gastrointest Liver Physiol. 2007;292: G1559-1569. doi:10.1152/ajpgi.00567.2006

46.Jijon HB, Suarez-Lopez L, Diaz OE, Das S, De Calisto J, Parada-Kusz M, et al. Correction: intestinal epithelial cell-specific RARα depletion results in aberrant epithelial cell homeostasis and underdeveloped immune system. Mucosal Immunol. 2019;12: 580. doi:10.1038/s41385-018-0074-8

47.Oehlers SH, Flores MV, Hall CJ, Crosier KE, Crosier PS. Retinoic acid suppresses intestinal mucus production and exacerbates experimental enterocolitis. Dis Models Mech. 2012;5: 457–467. doi:10.1242/dmm.009365

48.Bayeva N, Coll E, Piskareva O. Differentiating neuroblastoma: a systematic review of the retinoic acid, its derivatives, and synergistic interactions. J Pers Med. 2021;11: 211. doi:10.3390/jpm11030211

49.Melnik BC. Overexpression of p53 explains isotretinoin’s teratogenicity. Exp Dermatol. 2018;27: 91–93. doi:10.1111/exd.13420

50.Matthay KK, Reynolds CP, Seeger RC, Shimada H, Adkins ES, Haas-Kogan D, et al. Long-term results for children with high-risk neuroblastoma treated on a randomized trial of myeloablative therapy followed by 13-cis-retinoic acid: a children’s oncology group study. J Clin Oncol: Off J Am Soc Clin Oncol. 2009;27: 1007–1013. doi:10.1200/JCO.2007.13.8925

51.Makimoto A, Fujisaki H, Matsumoto K, Takahashi Y, Cho Y, Morikawa Y, et al. Retinoid Therapy for Neuroblastoma: Historical Overview, Regulatory Challenges, and Prospects. Cancers. 2024;16: 544. doi:10.3390/cancers16030544

52.Aguilar S, Louis C, Hicks J, Zage P, Russell H. Congenital neuroblastoma in a neonate with isotretinoin embryopathy. J Pediatr Hematol Oncol. 2014;36: e75-77. doi:10.1097/MPH.0b013e318290c39e

53.Humphrey HN, Sibley P, Walker ET, Keller DS, Pata F, Vimalachandran D, et al. Genetic, epigenetic and environmental factors in diverticular disease: systematic review. BJS open. 2024;8: zrae032. doi:10.1093/bjsopen/zrae032

54.Nehring P, Przybyłkowski A. Genetic determinants of colonic diverticulosis-a systematic review. Genes. 2025;16: 581. doi:10.3390/genes16050581

55.Yang Y, Chen Q, Zhong W. The role of cytokines in the pathogenesis of SAPHO syndrome. Front Immunol. 2024;15: 1427784. doi:10.3389/fimmu.2024.1427784

56.Wang Y, Gu M, Zheng Z, Jiang H, Han L, Huang H, et al. Therapeutic approaches for SAPHO syndrome from the perspective of pathogenesis: a review of the literature. Front Immunol. 2025;16: 1560398. doi:10.3389/fimmu.2025.1560398

57.Li SWS, Roberts E, Hedrich C. Treatment and monitoring of SAPHO syndrome: a systematic review. RMD open. 2023;9: e003688. doi:10.1136/rmdopen-2023-003688

58.Luzzati M, Simonini G, Filippeschi C, Giani T, Trapani S. SAPHO syndrome: the supposed trigger by isotretinoin, the efficacy of adalimumab and the specter of depressive disorder: a case report. Ital J Pediatr. 2020;46: 169. doi:10.1186/s13052-020-00933-1

4. **Comment:**

**Major Comments**

Reference Errors

Multiple entries throughout the manuscript display the placeholder “Error! Reference source not found.” This detracts from scientific credibility and should be resolved before further review.

**Reply:**

Dear Reviewer,

We sincerely apologize for the unacceptable presence of the “Error! Reference source not found.” placeholders throughout the manuscript. We thank the reviewer for meticulously identifying this critical issue, which understandably detracts from the scientific credibility of our work. This error occurred due to a failure in updating citation fields during a final document format conversion prior to submission.

We have taken immediate and thorough corrective action:

- We have regenerated the entire manuscript document using our reference management software (Zetero), ensuring all citation fields are properly linked to the bibliography database.
- We have performed a line-by-line manual verification of every in-text citation against the reference list to confirm accuracy and consistency.
- All placeholder errors have been resolved. The citations now appear correctly as numbered superscripts in the text (e.g., [1, 2]) and correspond accurately to the complete, formatted entries in the “References” section.
- We have ensured the reference list is complete and formatted strictly according to the journal’s guidelines.

We deeply regret this oversight and appreciate the opportunity to correct it.

5. **Comment:**

**Major Comments**

Methodological Clarity

While the statistical methodology is robust, the threshold definitions (Table 2) are overly technical. The authors should include brief plain-language explanations to improve accessibility to a broader audience.

**Reply:**

Dear Reviewer,

We sincerely thank the reviewer for the positive feedback on the robustness of our statistical methodology and for the valuable suggestion to improve its clarity for a broader readership. We agree that providing plain-language explanations of the technical thresholds is essential for enhancing the accessibility and impact of our research.

In direct response to your comment, we have made the following specific revision to the manuscript:

We have added a new explanatory paragraph immediately following Table 2 (which presents the formulas and threshold values). This paragraph provides a concise, non-technical interpretation of each of the four signal detection methods (ROR, PRR-MHRA, BCPNN, and MGPS) and their respective decision criteria. For instance, it explains that an ROR signal requires “at least 3 reports and a lower 95% confidence interval > 1, indicating a statistically significant increase in reporting odds,” and similarly clarifies the practical meaning of thresholds for PRR (PRR ≥ 2, χ² ≥ 4), BCPNN (IC-2SD > 0), and MGPS (EBGM05 > 2).

This addition directly translates the technical statistical criteria into their practical implications for pharmacovigilance signal detection, making the methodological section more transparent and understandable for clinicians, regulators, and researchers without specialized training in disproportionality analysis. We believe this revision successfully addresses your concern and improves the overall readability of the manuscript.

- **Please see below for the details: （Page 7-8, Line 363-399，the section highlighted in green）**

1.3.1 ROR method

This method evaluates the strength of association between a specific drug and an AE by calculating the ratio of reported events. According to standardized FDA criteria, a statistically significant potential safety signal is identified when the lower bound of the 95% confidence interval (95% CI) for the ROR is greater than 1, and the number of reports exceeds three.

1.3.2 PRR (based on the MHRA comprehensive criteria) method

The PRR method assesses drug-specific risk by comparing the proportion of a specific AE reported for the target drug with that reported for all other drugs. According to UK Medicines and Healthcare products Regulatory Agency (MHRA) criteria, a safety signal is considered present when the PRR is greater than 2, the χ² statistic exceeds 4, and the number of reports is at least three.

1.3.3 MGPS method

MGPS uses Bayesian statistical modeling to quantify associations between drugs and AEs. Signal strength is measured using the Empirical Bayes Geometric Mean (EBGM). A statistically significant safety signal is defined when the lower bound of the 95% CI of EBGM (EBGM05) is greater than 2.

1.3.4 BCPNN method

Based on Bayesian inference, the BCPNN method evaluates associations between drugs and AEs by calculating the Information Component (IC). According to established criteria, a potential safety signal is identified when the lower bound of the 95% CI of IC (IC–2SD) is greater than 0.

6. **Comment:**

**Major Comments**

Discussion of Teratogenicity and Pregnancy Events

The paper successfully highlights the persistent issue of unintended pregnancies during isotretinoin use. The discussion would benefit from a comparative overview of pregnancy prevention programs (e.g., iPLEDGE) and why such systems may fail, including real-world patient compliance data where available.

**Reply:**

Dear Reviewer,

Thank you for your positive feedback on our discussion regarding the teratogenicity of isotretinoin and the persistent challenge of unintended pregnancies. We agree that a deeper analysis of Pregnancy Prevention Programs (PPPs) and their real-world limitations would significantly strengthen the manuscript.

In direct response to your suggestion, we have substantially expanded the relevant section within the “Isotretinoin and pregnancy events” subsection of the Discussion. The revisions include:

**1.A Comparative Overview of PPPs:** We now explicitly contrast the stringent, centralized approach of the U.S. iPLEDGE program with the typically less rigid structures of some European PPPs, citing a dedicated comparative review [Kovitwanichkanont & Driscoll, 2018]. This contextualizes the discussion internationally.

**2.Analysis of System Failure Points:** We provide a structured, multi-level analysis explaining why PPPs may fail to prevent all pregnancies. This covers:

- Patient-level factors (e.g., risk underestimation, access barriers).
- System-level challenges (e.g., program complexity, digital divide).
- Provider-level burdens (e.g., administrative workload impacting counseling quality).

**3.Integration of Real-World Compliance Data:** We have more prominently and systematically integrated available real-world evidence on suboptimal patient compliance (e.g., adherence rates frequently below 50% and persistent pregnancy rates of 3.3%–6.5%) into this analytical framework to ground the discussion in empirical data.

These additions transform the section from a description of the problem into a critical examination of the systemic and behavioral factors that sustain it. We believe this greatly enhances the manuscript's value for clinicians and policymakers aiming to improve risk management strategies.

Thank you again for this constructive suggestion.

- **Please see below for the details: （Page 26-27, Line 1175-1223，the section highlighted in green）**

Given the unequivocal teratogenic risk of isotretinoin, numerous pregnancy prevention programs (PPPs) have been implemented worldwide. Among these, the U.S. iPLEDGE program is widely considered one of the most stringent regulatory strategies for managing risk [35,36]. Comparative studies have demonstrated substantial heterogeneity among PPPs internationally regarding regulatory rigor, contraceptive requirements, frequency of pregnancy testing, and enforcement mechanisms. In particular, iPLEDGE imposes a significantly greater administrative burden on both patients and healthcare providers [37].

Despite these measures, real-world evidence indicates suboptimal effectiveness of PPPs. Although such programs have existed for many years, approximately 3.3%–6.5% of isotretinoin users still experience pregnancy during treatment [38]. Contributing factors likely include inadequate adherence to contraceptive regimens, insufficient patient education on pregnancy prevention, and inappropriate selection of contraception methods. Population-based studies and registry analyses from the United States and Europe further indicate that, in routine clinical practice, adherence to key PPP components, such as contraceptive use, regular pregnancy testing, and enrollment procedures, is frequently poor, with complete compliance rates often below 50% [37,39-41]. Moreover, previous studies has reported that some patients fail to fully recognize the profound teratogenic risks associated with isotretinoin before starting treatment. This lack of understanding, combined with poor contraceptive adherence, contributes to persistently high unintended pregnancy rates [38].

The limited success of PPPs is multifactorial. Patient-level factors include inadequate health literacy, limited access to contraceptive resources, and socioeconomic barriers. System-level challenges involve procedural complexity, pharmacy accessibility, and difficulties navigating digital platforms. Additionally, provider-level issues such as workflow burdens and inconsistent counseling quality further undermine effectiveness [39-42]. Recent real-world cohort studies also show notably lower adherence among younger women and socioeconomically disadvantaged groups[40,42]. Collectively, these findings indicate that while programs like iPLEDGE have significantly reduced fetal exposure risks, they remain insufficient to completely prevent pregnancies. This highlights the necessity for ongoing optimization of pregnancy prevention strategies informed by real-world adherence data.

**References：**

35.Owens KE, Jiang SW, Nicholas MW. Contraceptive counseling for iPLEDGE and its burden on dermatologists. Arch Dermatol Res. 2024;316: 287. doi:10.1007/s00403-024-03070-5

36.Khiali S, Gharekhani A, Entezari-Maleki T. Isotretinoin; a review on the utilization pattern in pregnancy. Adv Pharm Bull. 2018;8: 377–382. doi:10.15171/apb.2018.044

37.Kovitwanichkanont T, Driscoll T. A comparative review of the isotretinoin pregnancy risk management programs across four continents. Int J Dermatol. 2018;57: 1035–1046. doi:10.1111/ijd.13950

38.Reinold J, Kollhorst B, Wentzell N, Platzbecker K, Haug U. Use of isotretinoin among girls and women of childbearing age and occurrence of isotretinoin-exposed pregnancies in Germany: A population-based study. PLoS Med. 2024;21: e1004339. doi:10.1371/journal.pmed.1004339

39.Ivask M, Kurvits K, Uusküla M, Juppo A, Laius O, Siven M. Compliance with Pregnancy Prevention Recommendations for Isotretinoin Following the Amendment of the European Union Pregnancy Prevention Program: A Repeat Study in Estonia. Drugs - Real World Outcomes. 2024;11: 91–98. doi:10.1007/s40801-023-00381-3

40.Brzeziński P, Feszak IJ, Śmigielski J, Kawczak P, Bączek T. Compliance with the European Pregnancy Prevention Programme in Isotretinoin Treatment: Safety Outcomes and Dose-Related Correlations. J Clin Med. 2025;14: 3497. doi:10.3390/jcm14103497

41.Choi EJ, Han JY. Non-compliance with pregnancy prevention recommendations for isotretinoin in korea between 2019-2020. Obstet Gynecol Sci. 2021;64: 201–208. doi:10.5468/ogs.20247

42.Havet A, Bouvard C, Moskal A, Chanelière M, Massardier J, Lebrun-Vignes B, et al. Compliance with the pregnancy prevention program among women initiating isotretinoin treatment between 2014 and 2021: A nationwide cohort study on the French Health Data System (SNDS). J Eur Acad Dermatol Venereol: JEADV. 2025;39: 806–814. doi:10.1111/jdv.20073

7. **Comment:**

**Minor Comments**

Please clarify whether the data extraction from FAERS was fully automated or manually curated after de-duplication.

**Reply:**

Dear Reviewer:

Thank you for raising this important methodological question, which helps enhance the transparency of our research pipeline. We fully agree that clearly stating the degree of automation and the nature of manual intervention in data processing is crucial for assessing the reproducibility and reliability of the study.

The data processing pipeline for this study can be described as “algorithm-driven with supplemental, limited manual quality control.” We have clarified this in the revised manuscript as follows:

Automated Core Pipeline: The extraction of isotretinoin-related reports from raw FAERS data subsets, the deduplication based on FDA-recommended algorithms (as described in section 1.2), and the mapping of adverse event terms to Medical Dictionary for Regulatory Activities (MedDRA) Preferred Terms (PTs) were all performed automatically using pre-programmed scripts in SAS 9.4 software to ensure consistency and reproducibility.

Manual Curation Component: Following this automated pipeline, we performed limited manual quality checks on the generated dataset. This primarily included: (a) cross-verification on random samples to ensure correct application of deduplication logic; (b) examination of records with outliers or logical inconsistencies (e.g., highly implausible patient age relative to the event); (c) verification of the accuracy of drug name and adverse event term mapping. This curation aimed to identify and exclude obvious anomalies potentially arising from data entry errors in the source or extreme technical artifacts, not to subjectively interpret or filter report content. All such manual interventions were documented and did not alter the core analytical variables generated by the automated process.

We have added the above clarification to the end of the “1.2 Data cleaning and standardization” subsection. We believe specifying this hybrid approach more accurately reflects the rigor of our study—leveraging automation for efficiency and consistency while employing limited manual curation to enhance the quality of the final dataset.

- **Please see below for the details: （Page 5, Line 291-301，the section highlighted in green）**

**Data processing workflow.** Data extraction and cleaning in this study were conducted primarily through automated procedures based on predefined algorithms to ensure objectivity and reproducibility. Specifically, identifying isotretinoin-related reports from FAERS subsets, deduplication procedures, and mapping of AE terms to MedDRA Preferred Terms (PTs) were all performed programmatically using SAS software (version 9.4). Upon completing the automated workflow, a manual quality control and verification step was applied. This step included logical validation of randomly selected data samples, inspection of extreme or clearly inconsistent values, and confirmation of drug–event term mappings. These manual checks aimed to detect and correct obvious anomalies, such as data entry errors, rather than to subjectively filter or reinterpret AE reports. All analyses were performed using the final dataset derived from this combined automated and manual quality verification pipeline.

8. **Comment:**

**Minor Comments**

In the “Limitations” section, explicitly state that incidence cannot be inferred from FAERS data.

**Reply:**

Dear Reviewer:

Thank you for this critical and important point. We fully agree with your comment. Explicitly stating that spontaneous reporting data from the US FDA Adverse Event Reporting System (FAERS) cannot be used to infer the incidence of adverse events is essential to prevent potential misinterpretation of the study findings by readers. This is a fundamental methodological limitation of pharmacovigilance signal detection studies.

Accordingly, we have added an explicit statement to the “4.2 Study Limitations” section of the manuscript (see detailed revision below). The statement clarifies that, due to the lack of a definitive exposed population denominator and consistent reporting completeness in FAERS data, the core value of this study—and similar signal detection studies based on such databases—lies in identifying “statistical association signals” for further investigation, not in quantifying the incidence or magnitude of risk for the adverse events.

We believe this addition significantly enhances the methodological rigor and transparency of the manuscript, ensuring its conclusions are framed within the correct interpretive context. We appreciate your help in improving our paper.

- **Please see below for the details: （Page 34, Line 1545-1551，the section highlighted in green）**

This study also cannot calculate or infer incidence, relative risk, or absolute risk of AEs. The FAERS database lacks reliable information on the number of exposed patients (denominator). Reporting behavior is influenced significantly by non-pharmacological factors such as medical practices, public awareness, media attention, and legal activities. This leads to substantial underreporting and reporting bias. Consequently, signal strength (e.g., ROR value) reflects statistical disproportionality, not the actual frequency or magnitude of risk. Signal mining serves to identify potential safety issues warranting further rigorous investigation (e.g., prospective cohort studies).

9. **Comment:**

**Minor Comments**

It would be useful to include a supplemental table summarizing the eight novel AE signals not previously included in the label, with frequencies and reporting years.

**Reply:**

Dear Reviewer,

Thank you for your constructive comment. We fully agree that a systematic summary of the eight novel adverse event (AE) signals not previously included in the isotretinoin product labeling would enhance the transparency and clinical relevance of our study.

In response, we have added a new supplemental table (S5 Table) in the Supporting Information section. This table provides a detailed overview of each novel signal, including the Preferred Term (PT) of reports in FAERS database, reporting years, Patient Gender and signal strength (ROR values). This summary facilitates a clear understanding of the epidemiological characteristics, temporal trends, and cross-database consistency of these signals.

We have referenced this table in the relevant paragraph. We believe this addition further strengthens the completeness and utility of our study.

We appreciate your valuable suggestion.

- **Please see below for the details:**

1. Specific details are shown in S1 and S5 Table.**（Page 10 , Line 456-457，the section highlighted in blue）**

2. S5_Table.Adverse Events from Top 30 Signals Not Documented in the Label in FAERS database.**（Page 42 , Line 1897-1898，the section highlighted in blue）**

10. ***Comment:***

***Recommendation:Major Revision***

*This is a comprehensive and potentially impactful pharmacovigilance study. However, improvements in the interpretation of key signals, discussion of biological plausibility, correction of formatting issues, and refinement of conclusions are necessary prior to publication.*

**Reply:**

Dear Editor,

Thank you for your positive assessment of our manuscript as a “comprehensive and potentially impactful pharmacovigilance study” and for providing constructive suggestions to further enhance its quality. We greatly appreciate the time and expertise invested in the review.

We have meticulously addressed all points raised. Specifically:

- Interpretation of Key Signals: We have substantially refined the discussion on critical signals, most notably for Inflammatory Bowel Disease (IBD). The revised text now includes a detailed analysis of temporal reporting patterns and reporter-type profiling, explicitly linking the signal spike to potential litigation-driven stimulated reporting, and contextualizing it with recent high-quality epidemiological evidence that disputes a causal association. This provides a much more balanced and critical interpretation (see revised sections in the Discussion, particularly under “Isotretinoin and AE signals related to IBD”).
- Biological Plausibility: For the novel signals not currently in the product label (e.g., nasal vestibulitis, anal papilla hypertrophy), we have expanded the discussion to incorporate potential mechanistic pathways based on the known pharmacology of retinoids (e.g., effects on epithelial differentiation, mucosal integrity, and immune modulation), supported by relevant preclinical and clinical literature citations (see “Biological plausibility and context of novel AE signals”).
- Formatting Issues: We have thoroughly checked and corrected formatting inconsistencies throughout the manuscript, including ensuring all tables and figures are correctly cited in the text, standardizing reference formatting, and verifying the accuracy of all statistical notations.
- Refinement of Conclusions: The Conclusion section has been rewritten to more accurately reflect the study's findings. It now emphasizes the distinction between statistical association and causation, highlights the need for cautious interpretation of signals like IBD, and clearly states that novel signals serve primarily for hypothesis generation, while reinforcing the paramount importance of pregnancy prevention.

Furthermore, to robustly validate the reliability and generalizability of our primary findings from the FAERS database, we have significantly expanded the external validation using the European EudraVigilance (EV) database. This was not explicitly requested but strengthens the manuscript considerably. The expanded analysis now provides a parallel, comprehensive signal detection in EV, including detailed demographic comparisons, SOC-level and PT-level signal concordance analyses, and a discussion on the reproducibility of findings across two major independent pharmacovigilance systems (see enhanced sections in “Result of external validation” and the corresponding updates in the Discussion and Conclusion).

We believe these comprehensive revisions have strengthened the manuscript's clarity, scientific rigor, and translational message. Thank you again for the valuable guidance. We hope the revised manuscript now meets the high standards for publication in your journal.

# Section 4: Responses to reviewer# 2

**(original comments are in yellow color)**

1. ***Comment:***

*The article is original: The manuscript presents a novel pharmacovigilance study analyzing 20 years of real-world adverse event.*

**Reply:**

Dear Reviewer：

We sincerely appreciate your acknowledgement of the originality of our pharmacovigilance study. This endorsement reinforces our commitment to further exploration of real-world data applications in drug safety surveillance.

2. ***Comment:***

*The study applies clear statistical formulas. Data cleaning, standardization and analysis steps are explicitly described. Results are presented with confidence intervals and comparative tables, supporting reproducibility.*

**Reply:**

Dear Reviewer：

Your affirmation of the significance of our results has been a source of great satisfaction and validation.

3. **Comment:**

The conclusions align with the presented data.

**Reply:**

Dear Reviewer：

Thanks for the positive comments.

4. **Comment:**

Language polishing: Revise for awkward or non-native phrasing, especially in the Introduction and Discussion sections (e.g., “strengthening preventive measures for pregnancy risks in patients is recommended” could be more concise and fluent). Consider professional English editing for clarity and tone consistency.

**Reply:**

Dear Reviewer,

Thank you very much for your valuable and meticulous feedback regarding the language quality of our manuscript. We fully agree with your assessment that ensuring conciseness, fluency, and tonal consistency is crucial for the clear communication of scientific findings. The example you provided (“strengthening preventive measures for pregnancy risks in patients is recommended”) aptly highlights an area where phrasing could be made more direct and idiomatic.

In direct and comprehensive response to your suggestion, we have engaged a professional English language editing service (proof of editing certificate is attached) to perform a thorough linguistic revision of the entire manuscript. This editing process was specifically focused on the Introduction and Discussion sections, as you highlighted, with the aim of eliminating any awkward or non-native phrasing and enhancing overall sentence clarity and flow.

For instance, the sentence you cited has been revised to a more concise and active form (see specific revision below). Beyond this example, the editing encompassed the standardization of terminology, optimization of sentence structures, strengthening of logical connectors, and harmonization of the academic tone throughout the text to align with the standards of leading international journals.

We are confident that this professional polish has significantly improved the readability and scholarly presentation of the manuscript. The revised version is now submitted for your review. Should any further refinements in language style or any other aspect be necessary, we are prepared to make additional modifications based on your expert guidance.

We sincerely appreciate your insightful comments, which have been instrumental in enhancing the quality of our work.

-**Please review the language editing certificate:**

5. ***Comment:***

*Clarify potential reporting bias: In the IBD section, the influence of legal-driven reports is well discussed. It would be helpful to include a short caution in the Conclusion to contextualize the strength of that signal.*

**Reply:**

Dear Reviewer,

Thank you for your valuable and highly constructive suggestion. We fully agree that including a contextual note and a word of caution in the Conclusion regarding the strong signal for inflammatory bowel disease (IBD) is crucial to ensure readers can interpret the findings correctly and in a balanced manner.

We have strictly followed your advice and incorporated an explicit statement in the Conclusion section (Section 5) of the manuscript. The newly added text is as follows:

“Notably, although inflammatory bowel disease (IBD) exhibited a strong statistical signal, reports were predominantly submitted by legal professionals from 2009 to 2014, indicating a potential litigation-driven reporting bias. Accordingly, interpretation of the IBD signal requires caution, as its magnitude may be substantially overestimated. Moreover, current high-quality clinical evidence supports that isotretinoin should not be considered an independent causal factor for IBD. ”**（Page 35 , Line 1609-1613，the section highlighted in green）**

Furthermore, to address your and another reviewer’s concerns regarding the cautious interpretation of the IBD signal more systematically and thoroughly, we used this opportunity to conduct a comprehensive revision of the relevant ‘Discussion’ section (3.4.2). The key enhancements include:

In-depth analysis of reporting bias: Beyond stating the proportion of lawyer-submitted reports (84.94%), we now provide a detailed explanation of their highly concentrated temporal pattern (85.75% occurring between 2009-2014) and its connection to litigation activities during that specific historical period, citing dedicated literature to support this as a classic example of “legal-driven over-reporting.”

Proactive integration of the latest clinical evidence: We proactively searched for and cited high-quality clinical studies published in recent years, including large-scale propensity score-matched cohort studies and the most recent meta-analysis. This evidence consistently indicates no independent causal relationship between isotretinoin and IBD after controlling for key confounders, providing solid counter-evidence for a cautious interpretation of the spontaneous reporting signal.

Through the in-depth argumentation in the Discussion section and the summarizing note in the Conclusion, we have established a more complete and rigorous framework aimed at guiding readers to fully appreciate the complexity behind the IBD signal. Your suggestion was a key part of this improvement process, and we thank you again.

- **Please see below for the details: （Page 23-25, Line 965-1081，the section highlighted in green）**

**3.4.2 Isotretinoin and AE signals related to IBD**

This study identified an exceptionally strong signal for inflammatory bowel disease (IBD), with a reporting odds ratio (ROR) of 579.14, ranking first among all detected signals and second in the number of reported cases, indicating an unusually high statistical strength. Given the extraordinary magnitude of the IBD signal, a detailed analysis of reporting patterns was conducted to better contextualize this finding.

In total, 5,278 IBD reports were identified, of which 4,483 (84.94%) were submitted by legal professionals. Temporal analysis further revealed a significant clustering of reports between 2009 and 2014, during which 4,526 cases (85.75%) occurred. In contrast, since 2018, fewer than 10 IBD reports have been submitted annually. This markedly skewed distribution in reporter type and reporting period strongly suggests non-random reporting dynamics.

To further elucidate the potential reasons for the strong isotretinoin-IBD association observed, the broader regulatory and legal context was examined. Following 2009, multiple high-profile lawsuits were filed in the United States against isotretinoin manufacturers, coinciding with a marked surge in IBD reports from 2009–2012. Previous investigations indicated that a substantial proportion of FAERS reports linking isotretinoin to IBD originated from law firms, highlighting potential litigation-driven stimulated reporting [24-25]. The reporting patterns observed in the present study closely align with known timelines of litigation activity in the United States. These results are consistent with previous studies documenting attorney-driven waves of isotretinoin-associated IBD reports in FAERS. Collectively, these findings indicate a period of litigation-driven overreporting likely occurred after 2009, and the exceptionally strong IBD signal detected here may largely reflect reporting bias.

Importantly, accumulating high-quality clinical evidence from recent years has consistently failed to demonstrate an independent association between isotretinoin exposure and IBD development. Large-scale population-based studies conducted over the past decade, along with updated meta-analyses and contemporary clinical guidelines, consistently suggest that IBD is predominantly driven by genetic susceptibility, environmental exposures, and immune dysregulation. These studies emphasize that such confounding factors frequently affect these patient populations, and that a direct causal relationship between isotretinoin and IBD has not been established[8,26-29]. Notably, large cohort studies employing propensity score–matched designs have shown that isotretinoin treatment, compared with appropriate acne-control populations, does not increase the risk of incident IBD [25,29]. Furthermore, several studies have reported that acne itself, rather than isotretinoin, is more strongly associated with subsequent IBD development, highlighting the critical role of confounding by indication in earlier observational findings. Systematic reviews and meta-analyses incorporating recent cohort data and trial sequential analysis further confirm no statistically significant association between isotretinoin use and IBD [26].

Collectively, although an exceptionally strong statistical signal for IBD was observed in this study, its clinical and pharmacological implications must be interpreted cautiously. Clinicians and regulatory authorities should remain attentive to shifts in reporting patterns, continuously integrate emerging high-quality clinical evidence, and provide scientifically grounded clarifications in drug labeling as appropriate. Such measures are essential for informed clinical decision-making and accurate public understanding of AE signals identified through pharmacovigilance systems.

**References：**

24.Stobaugh DJ, Deepak P, Ehrenpreis ED. Alleged isotretinoin-associated inflammatory bowel disease: disproportionate reporting by attorneys to the food and drug administration adverse event reporting system. J Am Acad Dermatol. 2013;69: 393–398. doi:10.1016/j.jaad.2013.04.031

25.Tenner S. Isotretinoin and inflammatory bowel disease: trial lawyer misuse of science and FDA warnings. Am J Gastroenterol. 2014;109: 570–571. doi:10.1038/ajg.2014.34

26.Yu C-L, Chou P-Y, Liang C-S, Chiang L-H, Wang T-Y, Tu Y-K, et al. Isotretinoin exposure and risk of inflammatory bowel disease: a systematic review with meta-analysis and trial sequential analysis. Am J Clin Dermatol. 2023;24: 721–730. doi:10.1007/s40257-023-00765-9

27.Kridin K, Ludwig RJ. Isotretinoin and the risk of inflammatory bowel disease and irritable bowel syndrome: a large-scale global study. J Am Acad Dermatol. 2023;88: 824–830. doi:10.1016/j.jaad.2022.12.015

28.Popescu CM, Bigby M. The weight of evidence on the association of isotretinoin use and the development of inflammatory bowel disease. JAMA Dermatol. 2013;149: 221–222. doi:10.1001/jamadermatol.2013.1348

29.Taylor MT, Margolis DJ, Kwatra SG, Barbieri JS. A propensity score matched cohort study identifying an association of acne, but not oral antibiotic or isotretinoin use, with risk of incident inflammatory bowel disease. J Am Acad Dermatol. 2023;88: 841–847. doi:10.1016/j.jaad.2023.01.014

6. ***Comment:***

*Improve figure and table referencing: Ensure all tables and figures (e.g., Fig 1–3, Table 1–5) are properly described and referenced in the main text with interpretation.*

**Reply:**

Dear Reviewer,

Thank you for your valuable comment regarding the improvement of figure and table referencing. We fully agree that ensuring all tables and figures are properly described and interpreted within the main text is essential for presenting data clearly and strengthening the logical flow of the manuscript.

In direct response to your suggestion, we have conducted a systematic check and revision of all figure and table references throughout the manuscript, with particular attention to the Results and Discussion sections. Our revision adhered to the principle that every citation of a figure (Fig 1–3) or table (Table 1–8, S1–S5) should be accompanied by at least a brief description summarizing its key finding or trend, rather than merely indicating its location.

We believe these revisions have significantly improved the clarity of data presentation and the cogency of the argument in the manuscript. The revised version is now submitted for your review. Should any further refinements be necessary, we are prepared to make additional modifications based on your guidance.

We sincerely appreciate your meticulous review.

7. ***Comment:***

*Add clinical implications: The authors might briefly discuss how clinicians can integrate this signal-mining approach into practice or pharmacovigilance systems, increasing the real-world impact of the findings.*

**Reply:**

Dear Reviewer,

We sincerely thank the reviewer for this insightful and constructive comment. We agree that discussing the clinical implications of signal-mining analyses is essential to enhance the real-world relevance of our findings.

In response, we have added a new subsection entitled “Clinical implications and integration of signal-mining findings into practice” in the Discussion section. In this subsection, we briefly outline how clinicians, hospital pharmacovigilance teams, and regulatory systems may integrate the identified and externally validated signals into clinical vigilance, safety monitoring programs, and structured signal management processes.

Specifically, we emphasize the role of reproducible high-ranking signals as early warning indicators that may inform enhanced patient counseling, targeted monitoring, and prioritization within pharmacovigilance systems, while reiterating that these signals represent associations rather than causality.

We believe that this addition meaningfully strengthens the translational value of our study and addresses the reviewer’s suggestion. The manuscript has been revised accordingly.

- **Please see below for the details: （Page 32, Line 1469-1490，the section highlighted in yellow）**

**3.5 Clinical implications and integration of signal-mining findings into practice**

Although disproportionality-based signal mining cannot establish causality at the individual patient level, it plays a critical role in contemporary pharmacovigilance by providing early warnings and prioritizing safety issues for further evaluation. The findings of this study may be integrated into clinical practice and pharmacovigilance systems in several ways.

First, clinicians may use high-ranking and reproducible signals identified across independent databases (FAERS and EudraVigilance) as triggers for enhanced clinical vigilance. For example, signals related to psychiatric disorders, pregnancy-related events, and rare but severe outcomes may prompt closer patient counseling, baseline risk assessment, and targeted follow-up during isotretinoin therapy, particularly in vulnerable populations.

Second, at the institutional level, hospital pharmacists and pharmacovigilance teams may incorporate such signal-mining results into local drug safety monitoring programs. Signals with high disproportionality and consistent external validation can be prioritized for periodic safety reviews, internal alerts, or integration into clinical decision support systems, thereby facilitating earlier detection of emerging safety concerns [59].

Finally, from a regulatory and systems perspective, signal-mining studies based on large spontaneous reporting databases support structured signal management processes, including signal validation, prioritization, and risk communication. When interpreted alongside clinical evidence and mechanistic plausibility, these data contribute to informed risk–benefit assessments and may guide updates to risk minimization measures or educational materials, as recommended in established pharmacovigilance frameworks [60].

**References：**

59. Potter E, Reyes M, Naples J, Dal Pan G. FDA Adverse Event Reporting System (FAERS) Essentials: A Guide to Understanding, Applying, and Interpreting Adverse Event Data Reported to FAERS. Clin Pharmacol Ther. 2025;118: 567–582. doi:10.1002/cpt.3701
60. Beninger P. Signal management in pharmacovigilance: a review of activities and case studies. Clin Ther. 2020;42: 1110–1129. doi:10.1016/j.clinthera.2020.03.018
