# Supplementary material for: Signal mining and analysis of adverse events associated with Isotretinoin: A 20-Year real-world pharmacovigilance study based on FAERS and EudraVigilance databases
Source: PLoS One. 2026 Jul 16;21(7):e0329161. doi: 10.1371/journal.pone.0329161 (PMC13483711; doi:10.1371/journal.pone.0329161)
Supplement: Peer review history

# Peer review: Decision Letter 0

**DOI:** 10.1371/journal.pone.0329161.r001

**Authors:** Gisele Viana de Oliveira

7 Aug 2025

PONE-D-25-37950

Signal Mining and Analysis of Adverse Events of Isotretinoin: 20-year real-world pharmacovigilance analysis based on the FAERS database

PLOS ONE

Dear Dr. Bian,

Thank you for submitting your manuscript to PLOS ONE. After careful consideration, we feel that it has merit but does not fully meet PLOS ONE’s publication criteria as it currently stands. Therefore, we invite you to submit a revised version of the manuscript that addresses the points raised during the review process.

==============================

**ACADEMIC EDITOR:**  The reviewers comments bellow should be fully addressed in your revised manuscript.

==============================

Please submit your revised manuscript by Sep 21 2025 11:59PM. If you will need more time than this to complete your revisions, please reply to this message or contact the journal office at plosone@plos.org. When you're ready to submit your revision, log on to https://www.editorialmanager.com/pone/ and select the 'Submissions Needing Revision' folder to locate your manuscript file.

Please include the following items when submitting your revised manuscript:

- A rebuttal letter that responds to each point raised by the academic editor and reviewer(s). You should upload this letter as a separate file labeled 'Response to Reviewers'.
- A marked-up copy of your manuscript that highlights changes made to the original version. You should upload this as a separate file labeled 'Revised Manuscript with Track Changes'.
- An unmarked version of your revised paper without tracked changes. You should upload this as a separate file labeled 'Manuscript'.

If you would like to make changes to your financial disclosure, please include your updated statement in your cover letter. Guidelines for resubmitting your figure files are available below the reviewer comments at the end of this letter.

If applicable, we recommend that you deposit your laboratory protocols in protocols.io to enhance the reproducibility of your results. Protocols.io assigns your protocol its own identifier (DOI) so that it can be cited independently in the future. For instructions see: https://journals.plos.org/plosone/s/submission-guidelines#loc-laboratory-protocols. Additionally, PLOS ONE offers an option for publishing peer-reviewed Lab Protocol articles, which describe protocols hosted on protocols.io. Read more information on sharing protocols at https://plos.org/protocols?utm_medium=editorial-email&utm_source=authorletters&utm_campaign=protocols.

We look forward to receiving your revised manuscript.

Kind regards,

Gisele Viana de Oliveira

Academic Editor

PLOS ONE

Journal Requirements:

When submitting your revision, we need you to address these additional requirements.

1. Please ensure that your manuscript meets PLOS ONE's style requirements, including those for file naming. The PLOS ONE style templates can be found at

https://journals.plos.org/plosone/s/file?id=wjVg/PLOSOne_formatting_sample_main_body.pdf and

https://journals.plos.org/plosone/s/file?id=ba62/PLOSOne_formatting_sample_title_authors_affiliations.pdf

2. We are unable to open your Supporting Information file “Supporting Information.zip”. Please kindly revise as necessary and re-upload.

3. We note you have included a table to which you do not refer in the text of your manuscript. Please ensure that you refer to Table 5 in your text; if accepted, production will need this reference to link the reader to the Table.

4. Please include captions for your Supporting Information files at the end of your manuscript, and update any in-text citations to match accordingly. Please see our Supporting Information guidelines for more information: http://journals.plos.org/plosone/s/supporting-information.

5. If the reviewer comments include a recommendation to cite specific previously published works, please review and evaluate these publications to determine whether they are relevant and should be cited. There is no requirement to cite these works unless the editor has indicated otherwise.

[Note: HTML markup is below. Please do not edit.]

Reviewers' comments:

Reviewer's Responses to Questions

**Comments to the Author**

1. Is the manuscript technically sound, and do the data support the conclusions?

The manuscript must describe a technically sound piece of scientific research with data that supports the conclusions. Experiments must have been conducted rigorously, with appropriate controls, replication, and sample sizes. The conclusions must be drawn appropriately based on the data presented.

Reviewer #1: Yes

Reviewer #2: Yes

**********

2. Has the statistical analysis been performed appropriately and rigorously?

Reviewer #1: Yes

Reviewer #2: Yes

**********

3. Have the authors made all data underlying the findings in their manuscript fully available?

The PLOS Data policy requires authors to make all data underlying the findings described in their manuscript fully available without restriction, with rare exception (please refer to the Data Availability Statement in the manuscript PDF file). The data should be provided as part of the manuscript or its supporting information, or deposited to a public repository. For example, in addition to summary statistics, the data points behind means, medians and variance measures should be available. If there are restrictions on publicly sharing data—e.g. participant privacy or use of data from a third party—those must be specified.

Reviewer #1: Yes

Reviewer #2: Yes

**********

4. Is the manuscript presented in an intelligible fashion and written in standard English?

PLOS ONE does not copyedit accepted manuscripts, so the language in submitted articles must be clear, correct, and unambiguous. Any typographical or grammatical errors should be corrected at revision, so please note any specific errors here.

Reviewer #1: Yes

Reviewer #2: Yes

**********

5. Review Comments to the Author

Please use the space provided to explain your answers to the questions above. You may also include additional comments for the author, including concerns about dual publication, research ethics, or publication ethics. (Please upload your review as an attachment if it exceeds 20,000 characters)

Reviewer #1: General Assessment

This manuscript addresses a highly relevant and timely topic in dermatology. Isotretinoin remains a cornerstone in the treatment of severe acne, yet its safety profile, particularly concerning psychiatric disorders, inflammatory bowel disease (IBD), and teratogenicity, continues to be the subject of debate.

The authors employ a robust methodological approach by integrating four pharmacovigilance signal detection methods (ROR, PRR, BCPNN, and MGPS) to analyze adverse events (AEs) associated with isotretinoin based on 20 years of data from the FAERS database.

The study contributes to the field by consolidating known risks and identifying eight potential new AE signals not currently included in the drug label. However, despite its merits, the manuscript requires major revisions prior to acceptance.

Major Comments

Causality vs. Association

The study relies on spontaneous reports, which inherently reflect association rather than causality. This critical distinction must be emphasized throughout the discussion and conclusion sections to avoid overinterpretation of signal strength.

Bias in IBD Reports

The authors acknowledge that over 84% of IBD reports were submitted by lawyers between 2009 and 2014, suggesting significant legal-driven reporting bias. While this is an important observation, the current framing may still overstate the strength of this signal. A more cautious interpretation is warranted.

New AE Signals

The inclusion of signals such as neonatal neuroblastoma, hypertrophic anal papilla, and hemihypertrophy is intriguing. However, these findings require more context regarding biological plausibility, previous literature, or mechanistic hypotheses before being suggested as candidates for label revision.

Reference Errors

Multiple entries throughout the manuscript display the placeholder “Error! Reference source not found.” This detracts from scientific credibility and should be resolved before further review.

Methodological Clarity

While the statistical methodology is robust, the threshold definitions (Table 2) are overly technical. The authors should include brief plain-language explanations to improve accessibility to a broader audience.

Discussion of Teratogenicity and Pregnancy Events

The paper successfully highlights the persistent issue of unintended pregnancies during isotretinoin use. The discussion would benefit from a comparative overview of pregnancy prevention programs (e.g., iPLEDGE) and why such systems may fail, including real-world patient compliance data where available.

Minor Comments

Please clarify whether the data extraction from FAERS was fully automated or manually curated after de-duplication.

In the “Limitations” section, explicitly state that incidence cannot be inferred from FAERS data.

It would be useful to include a supplemental table summarizing the eight novel AE signals not previously included in the label, with frequencies and reporting years.

Recommendation

Major Revision

This is a comprehensive and potentially impactful pharmacovigilance study. However, improvements in the interpretation of key signals, discussion of biological plausibility, correction of formatting issues, and refinement of conclusions are necessary prior to publication.

Reviewer #2: Some suggestions/considerations:

1-The article is original: The manuscript presents a novel pharmacovigilance study analyzing 20 years of real-world adverse event.

2-The study applies clear statistical formulas. Data cleaning, standardization and analysis steps are explicitly described. Results are presented with confidence intervals and comparative tables, supporting reproducibility.

3-The conclusions align with the presented data.

4- Language polishing: Revise for awkward or non-native phrasing, especially in the Introduction and Discussion sections (e.g., “strengthening preventive measures for pregnancy risks in patients is recommended” could be more concise and fluent). Consider professional English editing for clarity and tone consistency.

5- Clarify potential reporting bias: In the IBD section, the influence of legal-driven reports is well discussed. It would be helpful to include a short caution in the Conclusion to contextualize the strength of that signal.

6- Improve figure and table referencing: Ensure all tables and figures (e.g., Fig 1–3, Table 1–5) are properly described and referenced in the main text with interpretation.

7- Add clinical implications: The authors might briefly discuss how clinicians can integrate this signal-mining approach into practice or pharmacovigilance systems, increasing the real-world impact of the findings.

**********

6. PLOS authors have the option to publish the peer review history of their article (what does this mean?). If published, this will include your full peer review and any attached files.

If you choose “no”, your identity will remain anonymous but your review may still be made public.

**Do you want your identity to be public for this peer review?**  For information about this choice, including consent withdrawal, please see our Privacy Policy.

Reviewer #1: **Yes:** MARCO ALEXNADRE DIAS DA ROCHA

Reviewer #2: No

**********

[NOTE: If reviewer comments were submitted as an attachment file, they will be attached to this email and accessible via the submission site. Please log into your account, locate the manuscript record, and check for the action link "View Attachments". If this link does not appear, there are no attachment files.]

While revising your submission, please upload your figure files to the Preflight Analysis and Conversion Engine (PACE) digital diagnostic tool, https://pacev2.apexcovantage.com/. PACE helps ensure that figures meet PLOS requirements. To use PACE, you must first register as a user. Registration is free. Then, login and navigate to the UPLOAD tab, where you will find detailed instructions on how to use the tool. If you encounter any issues or have any questions when using PACE, please email PLOS at figures@plos.org. Please note that Supporting Information files do not need this step.

Attachment Submitted filename: Peer Review Report.docx

# Author comment: Author response to Decision Letter 1

**DOI:** 10.1371/journal.pone.0329161.r002

21 Feb 2026

Section 1�Response Letter

Dear Editor and Reviewers,

First and foremost, we would like to extend our sincere gratitude to you for your time and effort in reviewing our manuscript and providing us with such valuable, insightful, and constructive comments. Your feedback is of immense importance, offering crucial guidance not only for the revision and improvement of our current manuscript but also for inspiring our future research endeavors.

We have placed the highest priority on all the suggestions received. Upon receiving the review comments, our team engaged in thorough discussions and undertook a comprehensive and meticulous revision of the manuscript. We have addressed each point raised, leading to systematic enhancements in the methodological description, interpretation of results, depth of discussion, rigor of conclusions, as well as the language and formatting throughout the text. All modifications have been highlighted in color in the revised manuscript, and our detailed considerations along with specific locations of changes are provided in the point-by-point responses below.

It is important to note that we are fully aware of the current high standards in academic publishing, especially for studies based on real-world data mining, which demand robust and reproducible results. Therefore, despite the significant increase in data processing and analytical workload, we were committed to conducting a systematic external validation using the European EudraVigilance database. While not explicitly requested by the reviewers, we believe that demonstrating consistent findings across two independent, large-scale pharmacovigilance databases significantly strengthens the credibility and academic value of our conclusions. This additional work, however, substantially increased the complexity and time required for the revision process.

Our major revisions, undertaken in direct response to your key concerns, include the following:

1. Enhanced Methodological Transparency and Result Interpretation: We have added detailed descriptions of the data processing workflow and provided plain-language explanations of key statistical thresholds to improve readability and reproducibility.

2. Deepened Discussion and Cautious Interpretation: We have significantly strengthened the critical discussion of key signals (e.g., Inflammatory Bowel Disease), delving into potential reporting biases and integrating the most recent high-quality clinical evidence for balanced interpretation. A dedicated discussion on the biological plausibility of novel signals has been added.

3. Incorporation of External Validation for Robustness: We introduced a parallel analysis based on the EudraVigilance database, using cross-database consistency comparisons to further verify the reliability of our core findings.

4. Improved Formatting and Referencing: We have carefully checked and corrected all formatting issues per the journal's requirements, ensuring complete and accurate references, proper in-text citations for all tables and figures, and providing full captions for all Supporting Information files.

5. Expanded Clinical and Regulatory Implications: We added a new subsection discussing how signal-mining findings can be integrated into clinical practice and pharmacovigilance systems to enhance the real-world impact of the study.

6. Comprehensive Language Polishing: The entire manuscript has undergone professional English language editing to optimize clarity, fluency, and academic consistency.

At this point, we feel it is necessary to offer our most sincere apologies and provide a candid explanation for the delay in submitting this revision. The revision process was considerably challenged by the complexity of data processing and analysis, particularly due to the newly added external validation component. Compounding this, our team faced an unexpected personal hardship: the first author, who was primarily responsible for the core data analysis, experienced severe health challenges during this period due to a high-risk pregnancy, accompanied by exceptionally difficult physical reactions that necessitated periods of bed rest. Deeply committed to this research, she endeavored to resume work as soon as her condition slightly improved, striving to contribute to the revisions despite the circumstances. The confluence of these factors severely impacted our overall progress, leading to the regrettable delay. We fully understand and respect the timelines inherent to academic publishing and deeply regret any inconvenience this may have caused. We sincerely hope for your understanding regarding this exceptional situation. Despite the difficulties, we remained steadfast in our commitment to scientific rigor, valuing this critical revision opportunity immensely. We were determined to submit a manuscript that withstands scrutiny, refusing to compromise on the depth or quality of revisions due to time constraints.

We firmly believe that incorporating your expert insights and undertaking these additional efforts have significantly enhanced the scientific rigor, logical clarity, and academic value of the manuscript. Once again, we express our heartfelt thanks for your dedication, expert guidance, and kind understanding. We look forward to your further review and are prepared to fully cooperate with any subsequent modification requests.

Sincerely,

Yuan Bian

Section 2�Responses to Academic Editor

(original comments by Editor are in blue color)

1. Comment:

Please ensure that your manuscript meets PLOS ONE's style requirements, including those for file naming. The PLOS ONE style templates can be found at

https://journals.plos.org/plosone/s/file?id=wjVg/PLOSOne_formatting_sample_main_body.pdf and https://journals.plos.org/plosone/s/file?id=ba62/PLOSOne_formatting_sample_title_authors_affiliations.pdf

Reply:

Dear Editor,

Thank you very much for pointing this out. We fully understand and place high importance on adhering to the specific style requirements of PLOS ONE to ensure the smooth processing of our manuscript through the publication pipeline.

We have carefully reviewed the PLOS ONE style template links you provided and will strictly implement all relevant specifications in the revised manuscript. Our specific actions will include:

1.File Naming: We will ensure that all submitted files (main manuscript, figures, supplementary materials, etc.) follow the required naming conventions as per PLOS ONE's submission guidelines.

2.Template Application and Comprehensive Check: We will reformat our manuscript using the official templates (particularly the title/authors/affiliations template) as the baseline. This will encompass, but not be limited to:

• Adjusting the title page layout, author affiliation listing, and corresponding author designations.

• Standardizing the hierarchy and font formatting of section headings within the main text.

• Ensuring all figures, tables, and their legends comply with PLOS ONE's labeling and submission requirements.

• Converting both the in-text citation style and the end-of-text reference list format to the Vancouver style as required by PLOS ONE.

We confirm that these changes pertain solely to formatting and style and will not alter the scientific content, data, or conclusions of the paper. We appreciate your guidance in ensuring the manuscript meets the journal's standards.

2. Comment:

We are unable to open your Supporting Information file “Supporting Information.zip”. Please kindly revise as necessary and re-upload.

Reply:

Dear Editor,

Thank you for your note regarding the Supporting Information file. We apologize for the technical issue with the “Supporting Information.zip” archive and appreciate the opportunity to rectify it.

In response, we have thoroughly revised the Supporting Information in accordance with PLOS formatting guidelines. Specifically:

1. We have replaced the compressed archive with four individually named XLSX files (S1_Table.xlsx, S2_Table.xlsx, S3_Table.xlsx, S4_Table.xlsx�S5_Table.xlsx), each corresponding to the supporting tables referenced in the manuscript.

2. Each file is under 10 MB in size and contains complete statistical outputs from our disproportionality analyses in both the FAERS and EV databases.

3. We have updated the “Supporting Information” section in the manuscript.

These changes ensure that all supporting data are directly accessible without dependency on compression software, thereby aligning with PLOS recommendations for supporting information delivery.

The revised files have been uploaded to the submission system. Should any further adjustments be needed, we are happy to comply.Thank you for your guidance and support.

3. Comment:

We note you have included a table to which you do not refer in the text of your manuscript. Please ensure that you refer to Table 5 in your text; if accepted, production will need this reference to link the reader to the Table.

Reply:

Dear Editor,

Thank you for your careful review and constructive comment. We have noted the omission of the citation for Table 5 in the manuscript text. As suggested, we have now inserted a clear reference to Table 5 in Section 5.3 (“Classification of system organs involved in AE signals related to isotretinoin in the FAERS database”) where the SOC distribution and cumulative signal intensities are discussed.

Specifically, we revised the sentence to read:“Signal intensities for SOC-related AEs associated with isotretinoin are presented in Table 5.”

Furthermore, we have conducted a comprehensive check of all figure, table, and supporting information citations throughout the manuscript. We confirm that all other references (to Figures 1-3, Tables 1-4, 6-8, and S1-S4 Tables) are appropriately placed and meet the journal's formatting requirements.

This revision ensures that Table 5 is properly referenced, and our full self-audit guarantees overall consistency in citation, facilitating reader navigation and fully complying with journal production standards.

We appreciate your attention to detail and hope the manuscript now meets all required criteria.

• Please see below for the details: �Page 12 , Line 477-478�the section highlighted in blue�

Signal intensities for SOC-related AEs associated with isotretinoin are presented in Table 5.

4. Comment:

Please include captions for your Supporting Information files at the end of your manuscript, and update any in-text citations to match accordingly. Please see our Supporting Information guidelines for more information: http://journals.plos.org/plosone/s/supporting-information.

Reply:

Dear Editor,

Thank you for your valuable feedback regarding the captions for the Supporting Information files and the corresponding in-text citations. We greatly appreciate your clear guidance and have now duly revised the manuscript accordingly.

Specifically, we have:

1.Added complete captions for all Supporting Information files at the end of the manuscript, following PLOS formatting guidelines;

2.Updated the relevant in-text citations throughout the manuscript to ensure consistency.

These additions further improve the transparency and accessibility of the supporting data, helping readers better understand the relationship between the key findings presented in the text and the complete datasets provided.

The revised manuscript, which now includes the full Supporting Information captions and updated citations, has been re-uploaded to the submission system.

We believe these modifications fully address the points you raised. Should any further adjustments be needed, we are pleased to cooperate.

Thank you again for your review and support.

• Please see below for the details:

1. Specific details are shown in S1 and S5 Table.�Page 10 , Line 456-457�the section highlighted in blue�

2. Specific details are shown in S2 Table.�Page 12, Line 474-475�the section highlighted in blue�

3. Specific details are shown in S3 Table.�Page 17, Line 644-645�the section highlighted in blue�

4. Specific details are shown in S4 Table.�Page 18 , Line 661-662�the section highlighted in blue�

5. Supporing information�Page 41-42 , Line 1889-1898�the section highlighted in blue�

S1 Table. Statistical Analysis of PT Signals for Isotretinoin Adverse Events in FAERS database.

S2 Table. Statistical Analysis of SOC Involved in Adverse Event Signals of Isotretinoin in FAERS database.

S3 Table. Statistical Analysis of PT Signals for Isotretinoin Adverse Events in EV database.

S4 Table. Statistical Analysis of SOC Involved in Adverse Event Signals of Isotretinoin in EV database.

S5_Table.Adverse Events from Top 30 Signals Not Documented in the Label in FAERS database

5. Comment:

If the reviewer comments include a recommendation to cite specific previously published works, please review and evaluate these publications to determine whether they are relevant and should be cited. There is no requirement to cite these works unless the editor has indicated otherwise.

Reply:

Dear Editor,

Thank you for your valuable feedback. We fully acknowledge your note regarding the citation of previously published works. In this revision, we have carefully evaluated all references added during the modification process. Each citation was included solely based on its direct relevance to the methodological rigor, contextual background, or interpretive framework of our study. No citation was added due to external requests; rather, all references were selected to strengthen the scholarly foundation and transparency of our work. The added citations are appropriately placed in the Introduction, Methods, and Discussion sections as needed. We believe these additions enhance the academic integrity and clarity of the manuscript without unnecessary redundancy.

Thank you again for your review and support.

Section 3: Responses to reviewer# 1

(original comments are in green color)

1. Comment:

Major Comments

Causality vs. Association

The study relies on spontaneous reports, which inherently reflect association rather than causality. This critical distinction must be emphasized throughout the discussion and conclusion sections to avoid overinterpretation of signal strength.

Reply:

Dear Reviewer,

Thank you for your thorough review and valuable comments on our manuscript. You rightly pointed out that spontaneous reporting data reflect association rather than causality, which is a crucial distinction for enhancing the scientific rigor of this paper. We fully agree with your comment and have revised the Discussion and Conclusion sections accordingly to clarify the difference between “association” and “causality” and to avoid overinterpretation of signal strength.

Specifically, we have made the following revisions:

1. Added a paragraph at the beginning of the Discussion section to emphasize the associative nature of spontaneous reporting data.

• Please see below for the details: �Page 20 , Line 736-755�the section highlighted in green�

This study systematically analyzed AE signals related to isotretinoin using the FAERS spontaneous reporting system and four statistical methods. It is important to note that spontaneous reporting data only indicate statistical associations between drugs and AEs, not causality. Signal detection aims to identify potential risk signals to guide future clinical research, regulatory decisions, and clinical monitoring, rather than confirming direct causation. Therefore, the following discussion should be framed within the context of “association,” avoiding overinterpretation.

2. Incorporated cautious language in each key signal discussion subsection, explicitly stating that signals represent statistical associations only.

• Please see below for the details: �Page 23, Line 950-953�the section highlighted in green�

It should be noted that psychiatric signals detected in this study represent statistical associations only and do not imply direct causation by isotretinoin. Acne itself and its associated psychosocial stress may serve as confounding factors for psychiatric symptoms. Future prospective cohort or case-control studies are required to further investigate causality.

3. Further elaborated on the interpretative limitations due to data nature in the Study Limitations section.

Please see below for the details: �P

Attachment Submitted filename: Response to Editor and Reviewers.docx

# Peer review: Decision Letter 1

**DOI:** 10.1371/journal.pone.0329161.r003

**Authors:** Gisele Viana de Oliveira

11 Jun 2026

Signal mining and analysis of adverse events associated with Isotretinoin: A 20-Year real-world pharmacovigilance study based on FAERS and EudraVigilance databases

PONE-D-25-37950R1

Dear Dr. Bian

We’re pleased to inform you that your manuscript has been judged scientifically suitable for publication and will be formally accepted for publication once it meets all outstanding technical requirements.

Within one week, you’ll receive an e-mail detailing the required amendments. When these have been addressed, you’ll receive a formal acceptance letter and your manuscript will be scheduled for publication.

An invoice will be generated when your article is formally accepted. Please note, if your institution has a publishing partnership with PLOS and your article meets the relevant criteria, all or part of your publication costs will be covered. Please make sure your user information is up-to-date by logging into Editorial Manager at Editorial Manager® and clicking the ‘Update My Information' link at the top of the page. For questions related to billing, please contact billing support.

If your institution or institutions have a press office, please notify them about your upcoming paper to help maximize its impact. If they’ll be preparing press materials, please inform our press team as soon as possible -- no later than 48 hours after receiving the formal acceptance. Your manuscript will remain under strict press embargo until 2 pm Eastern Time on the date of publication. For more information, please contact onepress@plos.org.

Kind regards,

Gisele Viana de Oliveira

Academic Editor

PLOS One

# Editor report: Acceptance letter

**DOI:** 10.1371/journal.pone.0329161.r004

**Authors:** Gisele Viana de Oliveira

PONE-D-25-37950R1

PLOS One

Dear Dr. Bian,

I'm pleased to inform you that your manuscript has been deemed suitable for publication in PLOS One. Congratulations! Your manuscript is now being handed over to our production team.

At this stage, our production department will prepare your paper for publication. This includes ensuring the following:

* All references, tables, and figures are properly cited

* All relevant supporting information is included in the manuscript submission,

* There are no issues that prevent the paper from being properly typeset

You will receive further instructions from the production team, including instructions on how to review your proof when it is ready. Please keep in mind that we are working through a large volume of accepted articles, so please give us a few days to review your paper and let you know the next and final steps.

Lastly, if your institution or institutions have a press office, please let them know about your upcoming paper now to help maximize its impact. If they'll be preparing press materials, please inform our press team within the next 48 hours. Your manuscript will remain under strict press embargo until 2 pm Eastern Time on the date of publication. For more information, please contact onepress@plos.org.

You will receive an invoice from PLOS for your publication fee after your manuscript has reached the completed accept phase. If you receive an email requesting payment before acceptance or for any other service, this may be a phishing scheme. Learn how to identify phishing emails and protect your accounts at https://explore.plos.org/phishing.

If we can help with anything else, please email us at customercare@plos.org.

Thank you for submitting your work to PLOS ONE and supporting open access.

Kind regards,

PLOS ONE Editorial Office Staff

on behalf of

Dr. Gisele Viana de Oliveira

Academic Editor

PLOS One
